# Microglia-Astrocyte Crosstalk in Post-Stroke Neuroinflammation: Mechanisms and Therapeutic Strategies

**DOI:** 10.2174/011570159X350639250403072430

**Published:** 2025-04-25

**Authors:** Tong Shang, Binglin Kuang, Yaxin Shang, Wei Zou

**Affiliations:** 1 Heilongjiang University of Chinese Medicine, Heilongjiang, Harbin 150040, China;; 2 First Affiliated Hospital of Heilongjiang University of Chinese Medicine, Heilongjiang, Harbin 150040, China

**Keywords:** Ischemic stroke, hemorrhagic stroke, microglia, astrocytes, crosstalk, neuroinflammation, spatiotemporal dynamics, glia

## Abstract

Stroke is a leading cause of severe disability and mortality worldwide. Glial cells in the central nervous system (CNS) not only provide nutritional support but also play crucial roles in the inflammatory response. Microglia and astrocytes, integral components of the innate immune system, are involved in all stages of stroke and are active participants in inducing post-stroke neuroinflammation. Recent studies have increasingly focused on the potential crosstalk between microglia and astrocytes, identifying it as a promising area for understanding the pathogenesis and therapeutic mechanisms of CNS inflammatory diseases. These cells not only undergo dynamic phenotypic changes but also establish an intimate two-way dialogue by releasing various signaling molecules. This review paper elucidates the spatiotemporal dynamics of microglia and astrocytes in post-stroke neuroinflammation and highlights interaction pathways and potential therapeutic strategies for stroke.

## 1 INTRODUCTION

Stroke is an acute cerebrovascular disease that significantly threatens human health and quality of life worldwide. It ranks as the second leading cause of death globally, following ischemic heart disease, and is the third leading cause of death and disability [1]. In 2019, the incidence of stroke in China reached 276.7 cases per 100,000 population [2]. Stroke is categorized into hemorrhagic stroke (HS, including intracerebral hemorrhage and subarachnoid hemorrhage) and ischemic stroke (IS). IS is the most common type of stroke, accounting for 87% of all stroke cases and 62.4% of global new cases in 2019 [2].

Primary brain injury occurs immediately after stroke, essentially referring to mechanical brain damage, but it triggers a series of complex pathophysiological events, including cellular toxicity, neurotoxicity, oxidative and nitrosative stress, apoptosis, and inflammatory responses, leading to secondary brain injury (SBI) and further exacerbation [3]. Neuroinflammation is a crucial process during stroke, involving a cascade immune response mediated by cellular and molecular components. This includes the activation of microglia, astrocytes, and endothelial cells, as well as infiltration of peripheral immune cells and the release of inflammatory factors [4, 5]. Damage-associated molecular patterns (DAMPs) are molecules released by stressed or damaged cells, recognized by pattern recognition receptors (PRRs) as danger signals. This recognition triggers innate immune responses and initiates inflammatory cascades [6].

**Fig. (1):**
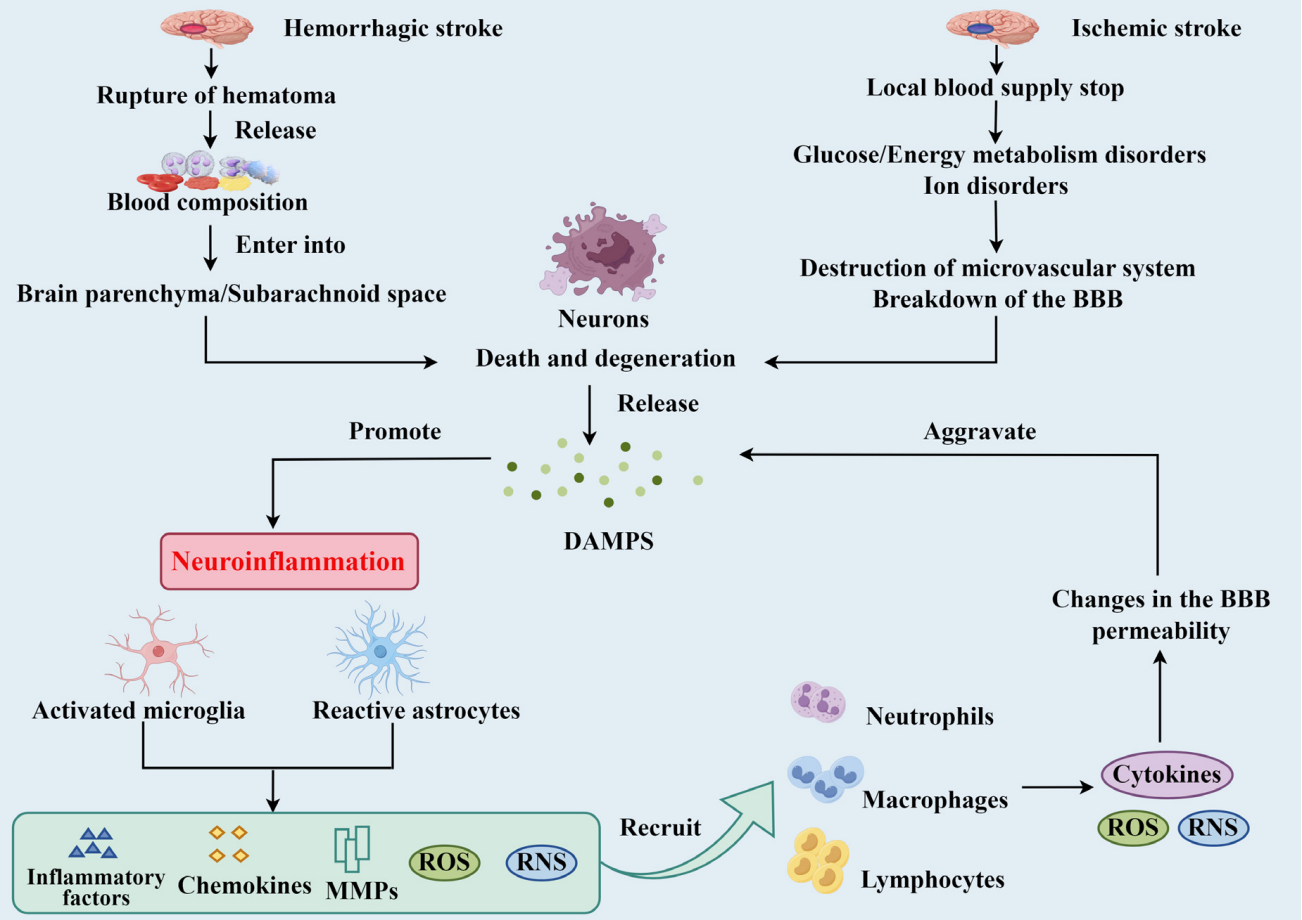
Sources of post-stroke neuroinflammation and inflammatory cascade. Both HS and IS produce DAMPs during the injury process, promoting immune-inflammatory responses in the brain and activating microglia and astrocytes. Activated microglia and reactive astrocytes release IL-1β, IL-6, IL-17, IL-18, tumor necrosis factor α (TNF-α), reactive oxygen species (ROS), reactive nitrogen species (RNS), and matrix metalloproteinases (MMPs). These induce wider activation of brain-resident cells and recruitment of peripheral cells, including neutrophils, monocytes (which mature into macrophages in the CNS), and lymphocytes. These inflammatory cells continuously produce cytokines, ROS, and RNS, changing the permeability of the BBB, forming a positive feedback loop of inflammation, and exacerbating brain damage. (Created by Figdraw).

In HS, DAMPs originate from blood components in the subarachnoid space and/or brain parenchyma and by-products of plasma proteins (thrombin, fibrinolytic enzymes, *etc*.). In IS, DAMPs arise from dead and degenerated neurons due to the microvascular system and the blood-brain barrier (BBB) disruption [7, 8]. DAMPs activate innate and non-immune cells, release cytokines and chemokines, and recruit more inflammatory cells. These cells, in turn, upregulate and actively secrete more DAMPs, forming a positive feedback loop that amplifies the inflammatory response. Ultimately, this cascade leads to brain edema, secondary brain ischemia, ischemic-to-hemorrhagic transformation, neuronal apoptosis, and various forms of irreversible brain damage, resulting in serious adverse outcomes (Fig. **1**) [9-11].

Microglia and astrocytes play extensive and critical roles in the development of the CNS. Under physiological conditions, they maintain highly bidirectional communication, crucially regulating aspects such as cell numbers, synaptic transmission, and vascular development [12, 13]. Microglia can regulate the proliferation of astrocytes, influence astrocytic control over neuronal dependence, and affect neurotransmission [14, 15]. Astrocytes, in turn, recruit microglia and promote physiological functions of microglia associated with synapses and neural circuits [16]. After stroke, microglia and astrocytes, as two major groups of reactive glial cells, become activated and polarized, exhibiting close interaction in neuroinflammation. Although previous studies have shown interactions and cascading amplification between microglia and astrocytes in neuroinflammation [17, 18], the mechanisms of crosstalk between them in post-stroke neuroinflammation require further elucidation. In this review, we discuss the pathophysiological changes of microglia and astrocytes after stroke, emphasizing the complex communication between these two cells in neuroinflammation and potential therapeutic strategies related to their crosstalk, providing valuable insights and targets for the pathological research and clinical treatment of stroke.

## 2 MICROGLIA AND POST-STROKE NEUROINFLAMMATION

### 2.1 Physiological Function of Microglia

Microglia originate from primitive myeloid progenitor cells within the yolk sac and migrate to the CNS during development, constituting 10-15% of neuroglial cells [19]. They maintain homeostasis of the CNS, phagocytize dead cells or cellular debris, participate in synaptic remodeling and pruning, regulate neuronal connections, and repair damage [20].

As regulatory cells in neuroimmune responses, microglia participate in inflammatory reactions and respond rapidly, acting as scavengers. In the resting state, microglia are inactive, with stationary cell bodies and highly branched processes that establish direct communication with other nerve cells and blood vessels for immune surveillance. However, when confronted with pathological stimuli, microglia become activated and respond swiftly to perform inflammatory functions. They transition from a highly branched morphology to amoeboid cells to cope with various challenges [21].

### 2.2 The Pathophysiology of Microglia in Post-Stroke Neuroinflammation

The response of microglia to different stages of stroke can be characterized by changes in their morphological function and polarization. Polarization is a phenotypic change in microglia induced by pathological stimulation, mainly including the “classically activated” M1 phenotype or the “selectively activated” M2 phenotype, which plays a dual role in the pro-inflammatory/anti-inflammatory and injury/repair processes [22]. Activation of M1 microglia promotes inflammatory response and leads to neurotoxicity, usually induced by lipopolysaccharide (LPS), Toll-like receptors (TLRs), and interferon-γ [23, 24]. Conversely, M2 microglia exhibit anti-inflammatory effects, participating in debris clearance and tissue repair after injury, and are usually induced by IL-4, IL-13, and TGF-β [25]. Apart from stroke, they are also well-represented in a wide range of neurodegenerative and neurotrauma-induced pathologies. Recent research has found that in addition to the classical pro- and anti-inflammatory phenotypes, there are more complex activation states, including dynamically balanced microglia expressing *Tmem 119* and *Hexb*, as well as other subtypes related to neural repair and stroke-associated microglia (SAM) with antioxidant properties [26, 27]. Although this binary classification is too simplistic due to the overlapping functional states and complex, evolved phenotypes of microglia, we continue to use the M1/M2 phenotype in this article for ease of understanding.

After stroke, microglia undergo temporal and spatial changes and can be activated and change their cellular morphology within minutes [28, 29]. During acute phases (<1 day), activation of microglia in the ischemic region first appeared around the lesion and correlated closely with the extent of neuronal damage. Thirty minutes after the onset of permanent middle cerebral artery occlusion (pMCAO), activated microglia were observed at the periphery of the ischemic lesion, characterized by enlarged cell bodies and thickened processes. Over time, microglia spread more widely and gradually extended into the cortex, peaking at 48 h [30]. Activation of microglia could be observed 3.5 h after MCAO reperfusion, whereas microglia in the ischemic core appear 12 h later [31].

In subacute and chronic phases, microglia undergo dynamic phenotypic changes. Perego *et al.* [32] observed in pMCAO that microglia showed different morphologies and functions at different time intervals. 24 h after ischemic injury, microglia were activated, showing a branched morphology in the peri-infarct region and an amoeboid morphology in the ischemic core. The rounded microglia were widespread in the ischemic area, likely corresponding to recruited peripheral immune cells. CD68 microglia were initially present at the border area and infiltrated the core area by 7 days, displaying characteristics of phagocytic cells that phagocyte neurons. Markers of M2 microglia (Ym1 and CD206) appeared abundantly in the ischemic core as early as 24 h after injury, with their protective effects persisting for at least 7 days before declining. In MCAO mice, researchers observed consistent expression levels of M1 microglial markers CD32 and pro-inflammatory cytokines (such as IL-1β and TNF-α), significantly increased by day 3, peaked at day 14, followed by a decrease and maintaining stability in the ischemic border area; whereas CD206 and Ym1/2 peaked at day 7 and declined rapidly thereafter [33]. Similarly, Hu *et al.* [34] found that in transient middle cerebral artery occlusion (tMCAO) mice, the gene expression level of M2 microglia was detected at 1-3 days after ischemia, peaked at 3-5 days, decreased at 7 days, and returned to the pre-injury level by day 14. In contrast, the gene expression of M1 microglia was initially low, starting to rise by day 3 and persisting until 14 days after ischemia. Shu *et al.* [35] also showed that microglia tended to be polarized into M2 phenotype 3 days after tMCAO.

However, in HS, microglia appear to exhibit an opposite polarization trend as in IS. During acute phases, the activation of microglia first occurred at 1 h after collagenase injection [36] and 4 h after autologous blood injection [37]. Activated microglia participate in hematoma phagocytosis. *In vitro* experiments indicate that a single microglial cell can phagocytize multiple red blood cells in a very short time, and with the increase of microglial density, multinucleated giant microglia/macrophages may appear around the hematoma and the surrounding area [38, 39]. In a collagenase-induced ICH model, Yang *et al.* [40] found that amoeboid microglia at the hemorrhagic center exhibited an M1 phenotype and selectively secreted IL-1β. Wan *et al.* [41] discovered that in ICH constructed by autologous blood injection, M1 microglia were activated early, appeared at 4 h, persistently highly expressed for 1-3 days, and declined after 7 days. In a collagenase-induced ICH model, Lan *et al.* [42] observed a significant increase in M1 (CD16/32^+^) microglia from 24 to 72 h, albeit with a reduced proportion due to a substantial increase in M2 (Ym-1^+^) microglia. In subarachnoid hemorrhage (SAH), Zheng *et al.* [43] observed a dynamic polarization process of microglia from M1 to M2 phenotypes, with morphological changes from ramified (1-3 days, M1) to spindle-shaped (bipolar microglia expressing both M1 and M2 markers) and finally to amoeboid shape (5-10 days, M2). Gris *et al.* [44] found in their study that IL-6 levels rapidly increased in both SAH patients and experimental SAH models.

**Fig. (2):**
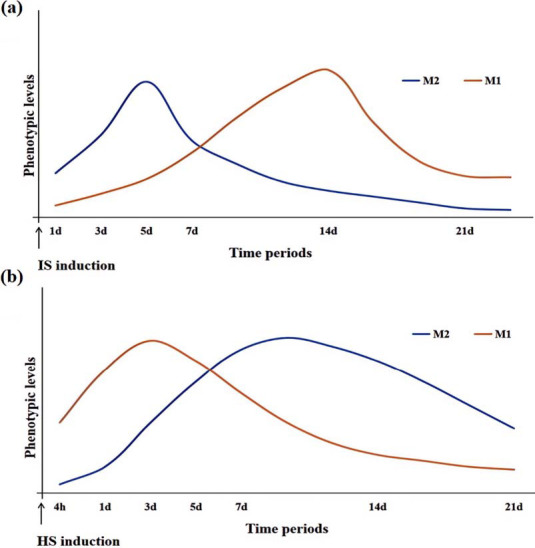
Dynamic changes in microglial polarization levels after IS and HS. (**a**) After IS, M1 microglia increase during the first 14 days and decrease rapidly. M2 microglia increase from day 1, peak on day 3-5, and return to pre-injury levels on day 14. (**b**) After HS, M1 microglia appear as early as 4 h after hemorrhage, which significantly increase within 3 days and subsequently decrease. M2 microglia begin to increase on day 1 after HS and persist for 1-2 weeks before decline. Evidence supports a switch of the M1 to M2 phenotype during the first 7 days.

In summary, the ratio of M1/M2 changes dynamically. Microglia transition from the dominant state of M2 to M1 in IS and M1 to M2 in HS (Fig. **2**). This also reflects the difference between HS and IS in neuroinflammation, that is, in the early stage inflammatory response is heavier in HS, while ischemia and hypoxia is heavier in IS. Although there is an inflammatory response in IS, the main role may be to promote repair and neuroprotection. With the increase of time and the accumulation of inflammatory factors, neuroinflammation will further aggravate. However, to date, the exact timing and triggers of the phenotypic changes remain unknown. Different models of stroke, the degree of bleeding/infarction, and the size of the lesion are all factors affecting M1 and M2 polarization. In addition, aging gender has also been shown to influence phenotypic changes [45, 46]. Therefore, it is important to assess the time course of M1 and M2 polarization after stroke to further investigate the timing of the most efficient transition to phenotypes, in order to select the optimal temporal switch to reduce inflammation and improve neurological outcomes.

## 3 ASTROCYTES AND POST-STROKE NEUROINFLAMMATION

### 3.1 Physiological Function of Astrocytes

Astrocytes originate from glial cells of neural precursors and are the most abundant in the CNS, accounting for approximately 20-40% of glial cells [47]. In the past, astrocytes were considered as a “glue-” like function, providing structural and nutritional support for neurons. In recent years, more and more functions have been elucidated. Astrocytes are the main cells in the neurovascular unit and are essential for the formation and growth of cerebrovascular beds and the maintenance of neurovascular function [48]. Astrocytes regulate the release of active molecules such as glutamate (Glu) and adenosine and modulate the excitability and synaptic transmission of neurons. They also control the strength and plasticity of synapses through gap junctions and provide cellular connections between neuronal circuits and blood vessels, which are helpful in regulating local blood flow, maintaining the dynamic balance of extracellular fluid, responding to neuronal activities, and coordinating glucose metabolism [49].

### 3.2 The Pathophysiology of Astrocytes in Post-Stroke Neuroinflammation

After stroke, under the stimulation of microglia and inflammatory factors, astrocytes are activated and then rapidly proliferate and become hypertrophic, leading to reactive astrocytosis. This is a graded reaction characterized by the increased expression of intermediate filament proteins, including glial fibrillary acidic protein (GFAP), vimentin, and nestin [50]. GFAP is a structural protein, and its expression changes are associated with the proliferation intensity of reactive astrocytes. Within the time window of 2-6 h after stroke, GFAP levels can distinguish between IS and early ICH [51]. In the early stages of IS, aquaporins (AQPs) regulate the rapid membrane expansion and transport of water, causing astrocytes to swell, which leads to the increase of intracranial pressure and the decrease of cerebral blood flow [52]. After HS, blood extravasation activates the coagulation cascade to produce thrombin, which not only leads to the formation of perihematoma edema but also triggers the sustained activation of protease-activated receptor PAR1 in astrocytes. This activation induces rapid remodeling of astrocyte processes adjacent to glutamatergic synapses, including contraction, flattening, proliferation, and foot processes away from excitatory glutamatergic synapses, potentially affecting long-term neuroplasticity and exacerbating brain injury [53].

Reactive astrocytes play a dual role at different stages of stroke. On one hand, they release a large number of inflammatory factors, MMPs, and chemokines to aggravate inflammation and induce neuronal death; on the other hand, they release trophic factors, lipoxins A4 and B4, excitatory amino acids to inhibit neuroinflammation, and regulate the production of microglial inflammatory mediators to promote neuroprotection, exert anti-inflammatory effects and promote synaptic formation and neuronal survival [54, 55]. After stroke, reactive astrocytes usually present two subtypes: the pro-inflammatory A1 phenotype and the anti-inflammatory A2 phenotype. With the deepening of research, the phenotypes of astrocytes are gradually enriched, and according to their differences in gene expression, they can present a variety of activation states from pro-inflammatory to anti-inflammatory rather than simple A1 and A2 polarized phenotypes [56]. Therefore, we used a pro-inflammatory/anti-inflammatory description in place of the A1/A2 phenotype.

After stroke, astrocytes preferentially differentiate into a “pro-inflammatory” phenotype rather than an “anti-inflammatory” phenotype. In the tMCAO mice, within 14 days, the number of perifocal regions C3d^+^/GFAP^+^ cells in the marginal area of ischemic focus increased gradually, and the expression of inflammatory factors such as TNF and IL-6 increased. On day 3 after the stroke, a large number of pro-inflammatory astrocytes were wrapped around the blood vessel wall and destroyed the integrity of the BBB [57]. In MCAO, the number of pro-inflammatory astrocytes in the penumbra of cerebral ischemia increased continuously to the peak on day 14, and the appearance of anti-inflammatory astrocytes was delayed, peaked on day 7, and decreased on day 14 [58]. In the tMCAO model, researchers found that after 72 h, the anti-inflammatory-related transcription predominated over the pro-inflammatory-specific transcripts, suggesting neuroprotective effects [59]. After ICH, the pro-inflammatory astrocyte marker C3 was active and peaked on day 3 [60]. After experimental SAH, the continuous increase of C3 level indicated that astrocytes preferentially differentiated into a pro-inflammatory phenotype [61, 62]. Obviously, the trend of morphology and phenotype of astrocytes after HS and IS is consistent in time and space, but the transformation and peak time of pro-inflammatory/anti-inflammatory phenotype may be affected by different models and disease severity.

Within days after injury, around the lesion, activated astrocytes further form glial scar by secreting extracellular matrix molecules to participate in the regulation of neuroinflammation. Glial scar is a network formed by glial cell processes and thin filament proteins in plasma, whose formation is highly dependent on hypertrophy, proliferation, and cellular overlap of microglia and astrocytes [63]. Finally, abundant proliferating astrocytes form glial scars between damaged areas and healthy tissue. In the early stages, it reduces excitotoxicity by siphoning K^+^ and Glu uptake. By forming physical and functional walls around the lesion, the glial scar closes the injured site, regulates the plasticity of nerves, and controls the local immune response in space and time to inhibit the spread of inflammation [64]. However, in the late stages, the uncontrolled proliferation of glial scars releases pro-inflammatory factors and expresses a series of molecules that inhibit neuronal migration and axonal regeneration, such as chondroitin sulfate proteoglycans (CSPGs), which is not conducive to nerve regeneration [65]. Therefore, the heterogeneity of phenotype and function of reactive astrocytes after stroke affects the progress of inflammation.

## 4 MICROGLIA-ASTROCYTE CROSSTALK PROMOTES POST-STROKE NEUROINFLAMMATION

### 4.1 Microglia Activate Pro-Inflammatory Astrocytes

Microglia are more sensitive to pathogens/damage, whereas astrocyte responses are usually delayed and cannot be fully activated in the absence of microglia [66]. After a stroke, microglia preferentially respond to pathological stimuli and secrete IL-1α, TNF-α, and C1q after activation, triggering reactive signals of astrocytes through soluble and membrane-bound signaling molecules, inducing their transformation into pro-inflammatory astrocytes. This leads to neuronal and oligodendrocyte death and forms a neuroinflammatory cascade [54, 67, 68]. Whether through acute CNS injury or systemic LPS injection, microglial activation induces pure pro-inflammatory astrocytes both *in vitro* and *in vivo* [67]. C1q is one of the initiating molecules of the classical complement activation pathway, which produces chemotactic C3a and C5a through the complement cascade. On one hand, this attracts neutrophils and monocytes to migrate to the lesion and activates cells to release more inflammatory mediators. On the other hand, it activates astrocytes toward a pro-inflammatory phenotype and contributes to synaptic toxicity [69, 70]. Astrocytes express the class F scavenger receptor Megf10, a receptor for C1q, and can clear apoptotic neurons through the Megf10/C1q pathway [71]. However, under pathological conditions, C1q levels rise sharply, temporarily increasing ROS and NO levels, promoting pro-inflammatory cytokines, and leading to neuronal death [72]. Additionally, astrocytes release C3a, which binds to the C3a receptors (C3aR) on microglia, thereby triggering microglial secretion of more C1q and pro-inflammatory factors [73]. Deleting C1q in microglia not only prevents the deposition of C3a on astrocytes but also reduces the upregulation of TLR4 in microglia [74]. Activated microglia also release interleukin, monocyte chemotaxis protein-1 (MCP-1), and macrophage colony-stimulating factor (M-CSF), which is crucial in the initial triggering and regulation of astrocytes during acute phases [75].

TNF-α and IL-1 not only act as inflammatory factors to trigger reactive astrogliosis but also act as a pro-inflammatory signal amplifier in microglia-astrocytes crosstalk, amplified by the unique physiological structure of astrocytes. In experimental stroke models, TNF-α and IL-1 were overexpressed as early as 2 h after ICH and 24 h after IS [76, 77]. Astrocytes express IL-1β receptor (IL-1R) [78], and IL-1 stimulates the expression of GFAP and hypertrophy of astrocytes, indicating its important role in mediating astrocyte activation [79]. TNF-α receptors exist in astrocytes, and their activation can inhibit the excitatory amino acid transporter (EAAT) on astrocytes, causing increased extracellular Glu concentrations and neurotoxicity, promoting calcium influx and cellular overload. Simultaneously, they activate microglial-mGluR2, promote further release of TNF-α, and exacerbate inflammatory response [80, 81].

SDF-1α, also known as CXC chemokine ligand 12 (CXCL12), and its receptor CXCR4 have been reported to be upregulated in cerebral ischemic penumbra tissue, which attracts inflammatory cells to release pro-inflammatory factors and aggravate brain injury [82, 83]. SDF-1α is a known stimulator of astrocyte proliferation [84]. Studies have confirmed that astrocytes are involved in SDF-1α/CXCR4 autocrine/paracrine signaling [85]. After a stroke, TNF-α amplifies the Glu release cascade through SDF-1α/CXCR4, increases neuronal excitotoxicity, promotes the continuous release of pro-inflammatory mediators (TNF-α, IL-1β, and IL-6) from microglia and astrocytes, and positively feedback regulates the neuroinflammatory microenvironment [86, 87]. The release of TNF-α stimulates microglia to express high levels of HMGB1 and TLR4, increases inflammatory cytokines and NF-κB activity, and promotes the response of astrocytes [88].

In addition to pro-inflammatory cytokines, mitochondria may become a new mechanism of glial cell interactions in neuroinflammation. Mitochondrial fission and fusion, as the basis of mitochondrial quality control, regulate the activity and function of glial cells, ensuring the dynamic equilibrium state of mitochondria in response to changes in stress conditions [89]. After the stroke, excessive mitochondrial fission triggers apoptosis, which is mediated by dynamin-associated protein 1 (Drp1). The key process is the phosphorylation of Drp1 and its subsequent relocalization to the outer mitochondrial membrane and binding to the Fis1 receptor, which subsequently promotes microglia-induced neuroinflammation [90, 91]. Mitochondrial morphology becomes discontinuous after accelerated fission, leading to mitochondrial fragmentation, and these microglial extracellular mitochondria activated by DAMPs are a driving force for the progression of secondary neuronal damage, participating in communication with astrocytes [92]. Recent research showed that under pathological conditions, DAMPs and neurotoxic proteins further activated microglia, induced excessive mitochondrial fission mediated by Drp1/fis1, and mitochondrial dysfunction. This released fragmented extracellular mitochondria and generated diffusion signals to astrocytes into a neurotoxic phenotype, exacerbating pathogenic inflammation [93]. Liu *et al.* [94] found that in tMCAO, M1 microglia released damaged and unhealthy mitochondrial debris, transferred to neurons, and fused with neuronal mitochondria, deteriorating neurological prognosis. To date, the potential interaction of intercellular mitochondrial transmission in glia-glia and glia-neuron after stroke remains unclear and warrants further investigation.

### 4.2 Activated Astrocytes Induce M1 Microglia

Astrocytes activated by microglia lose many physiological functions and release inflammatory cytokines, chemokines, ATP, and specific proteins that act on microglia, thus forming a paracrine feedback loop that aggravates neuroinflammation.

#### 4.2.1 IL-17A/IL-17RA

IL-17A and its receptor IL-17RA are involved in post-stroke inflammation and disruption of the BBB. IL-17A belongs to the IL-17 family (IL-17A~IL-17F), which is a major inflammatory mediator and can induce the expression of inflammatory genes [95]. Astrocytes are the main source of IL-17A production. During acute phases of stroke, IL-17A is involved in microglial activation and neuroinflammation, promoting the expression of TNF-α, IL-1β, and other inflammatory factors and downstream signaling molecule NF-κB p65 [96, 97]. IL-17A also induces chemokines CXCL1 and CXCL2 to participate in the expansion and recruitment of neutrophils, which aggravates neuroinflammation [98, 99]. IL-17RA exists on the surface of microglia and is the main target of the IL-17 signaling pathway [100, 101]. Studies have shown that the knockdown of IL-17A significantly reduces the activation of microglia and promotes their transformation to the M2 phenotype [102]. Therefore, the IL-17A/IL-17RA pathway induces the formation of M1 microglia after stroke.

#### 4.2.2 IL-15/IL-15R

IL-15 is a cytokine that regulates the proliferation, chemotaxis, and survival of immune cells. In the inflammatory CNS, IL-15 is mainly derived from astrocytes [103, 104]. IL-15 is a factor specifically upregulated after stroke and acts as an immune enhancement regulator. Studies have shown that astrocyte-derived IL-15 in the ischemic brain promotes the migration of T cells to inflammatory tissues, affects the activation and effector function of CD8^+^ T cells and NK cells, aggravates brain damage, and knockdown of IL-15 improves the immune response of the damaged brain [103, 105, 106]. In addition, IL-15 can enhance the Th1 response after acute cerebral ischemia, which is manifested by an increase in the number of CD4^+^ T cells [105]. The IL-15 receptor (IL-15R) is a heterotrimeric receptor composed of three chains: IL-15Rα, IL-2/IL15Rβ, and γc [107]. Intracellular IL-15 transmits signals through a “trans-presentation” mode, binding to high-affinity IL-15Rα to form a complex, triggering signal transduction through IL-15Rβ and γc on neighboring cells [108]. Microglia express IL-15R and thus could be receptive to IL-15 from astrocytes to form crosstalk [109]. Recent studies have shown that IL-15 plays a mediator in microglia-astrocyte crosstalk after ICH, increasing the expression of CD86, IL-1β, and TNF-α from microglia and skewing microglia toward a pro-inflammatory phenotype. Astrocyte-derived IL-15 mainly affects the response of microglia. After the elimination of microglia, the aggravation of brain injury caused by IL-15 is attenuated [110].

#### 4.2.3 CCL2/CCR2

Chemokine ligand 2 (CCL2), also known as MCP-1, is the main endogenous agonist of CCR2 and is involved in the inflammatory response after CNS injury. In neuroinflammation, CCL2 is mainly derived from astrocytes and can bind to CCR2 to induce microglial activation [111, 112]. After stroke, CCL2/CCR2 induces microglial recruitment, increases leukocyte infiltration and the expression of inflammatory mediators, further aggravates damage to the BBB, and leads to brain edema and neuronal death [113, 114]. Microglia express CCR2 [115]. In *in vitro* cell culture experiments, TNF-α stimulation causes astrocytes to release a large amount of CCL2, which enhances the ability of microglia to migrate to the site of injury through the CCL2/CCR2 pathway and induces their polarization to the M1 phenotype. The above phenomenon is inhibited after CCL2 siRNA or the use of CCR2 inhibitors [116].

#### 4.2.4 GM-CSF/GM-CSFR

Granulocyte-macrophage colony-stimulating factor (GM-CSF) is mainly produced by astrocytes, which is a potent activator of microglia and participates in the pro-inflammatory process [111, 117]. GM-CSF exerts its biological function by binding to the GM-CSF receptor complex. GM-CSFR is highly expressed in microglia in the CNS [118]. GM-CSF can cross the BBB and may mediate the pathogenic effects of neuroinflammation [119]. Although GM-CSF itself can not induce the secretion of classic inflammatory cytokines like IL-1β or TNF-α, it can be achieved by acting directly or indirectly on microglia. GM-CSF promotes microglial migration through the upregulation of cathepsin, MMP-9, -11, and -12, thereby triggering excessive inflammatory responses [120]. Studies have shown that GM-CSF increases the expression of TLR4 and CD14 in microglia by activating ERK1/2 and p38 and promotes neuroinflammation by increasing the production of LPS-induced inflammatory mediators (IL-1β, IL-6, TNF-α, NO) [118]. In a microglia-astrocyte co-culture system, GM-CSF stimulates the proliferation of microglia *in vitro* and induces polarization of M1 microglia [121].

#### 4.2.5 S100B Protein

S100B, a member of the S100 protein family, is predominantly expressed in astrocytes in the brain. It is an alarm protein released during brain injury and acts as an intracellular regulator and extracellular signal and participates in various cellular processes such as cell proliferation, differentiation, apoptosis, inflammation and metabolism [122, 123]. Under physiological conditions, S100B has trophic effects on neurons and promotes Glu uptake. However, it increases after stroke, inducing migration and morphological changes of microglia, upregulating the expression of pro-inflammatory transcription factors, and stimulating the release of MMP-9 and NO, leading to cytotoxic effects and SBI [124]. Extracellular S100B may upregulate chemokine expression through RAGE and stimulate microglial migration, thus participating in the dissemination of inflammation in the brain [125]. Zhou *et al.* [126] showed that in MCAO, S100B induced excessive production of INOS and high-level release of NO, activated NF-κB, promoted the polarization and migration of M1 microglia, and aggravated inflammatory damage after cerebral ischemia. The study found that the administration of Arundic Acid (AA) to inhibit S100B could prevent the increase in microglial activation after injury [127].

#### 4.2.6 Connectin 43

Connexin 43 (Cx43), composed of gap junction and hemichannel functional units, is one of the main gap junction proteins in astrocytes, involved in maintaining cellular and tissue homeostasis [128]. In the early stages of stroke, Cx43 is involved in indirect interactions between astrocytes and microglia, mediated through a vicious cycle involving Cx43 hemichannel activation. On one hand, activated microglia release pro-inflammatory factors (such as TNF-α and IL-1β), increasing the permeability of astrocytic Cx43 hemichannels, leading to the release of ATP, Ca^2+^, and Glu, and regulating downstream signaling intermediates (including STAT3, p38 MAPK, NF-κB), thereby enhancing the proliferation of reactive astrocytes and activation of M1 microglia, intensifying pro-inflammatory activity [129, 130]. On the other hand, ATP can activate purinergic receptors, known as P2 receptors, to enable the continuous activity of the inflammasome in various brain cell types, including microglia, promote the spread of ischemic brain injury, and aggravate neuroinflammatory injury [131]. After a stroke, P2X7 receptors on microglia are activated by ATP, triggering the release of IL-1β, IL-18, and TNF-α, promoting apoptosis and contributing to deep ion imbalance during neuronal death [132, 133]. Recently, researchers have developed genetically encoded fluorescent sensors for monitoring the spatiotemporal dynamics of ATP *in vivo*. Results indicated functional specialization and coordination between astrocytes and microglia, with astrocytes sensing damage and encoding injury information and microglia subsequently decoding this information to adapt to various stimuli and drive changes in their activation states [134].

#### 4.2.7 PRDX6-iPLA2

Peroxiredoxin 6 (PRDX6) is the sole 1-Cys member of the peroxiredoxin family, functioning as a dual enzyme with glutathione peroxidase and Ca^2+^-independent phospholipase A2 (iPLA2) activities. Increasing evidence suggests that PRDX6 is predominantly expressed in astrocytes [135, 136]. In primary cultured astrocytes, the iPLA2 activity of PRDX6 induces its own proliferation and increases pro-inflammatory factors [137]. Shanshan *et al.* [138] observed in OGD/R co-cultures of microglia/neurons and in the MCAO model that PRDX6-iPLA2 is associated with the secretion of neurotoxic inflammatory mediators in microglia, potentially regulating neuroinflammation *via* TLR2/4. Downregulation of PRDX6-iPLA2 significantly mitigates neuronal damage in co-cultures. Recently, Peng *et al.* [139] found that after cerebral ischemia-reperfusion injury, PRDX6-iPLA2 activity is significantly upregulated in astrocytes, inducing ROS production and further promoting M1 microglial polarization, revealing the mechanisms of astrocyte-microglia crosstalk and oxidative stress-neuroinflammation crosstalk. Future research should explore how PRDX6 serves as a multifaceted communicator between astrocytes, neurons, microglia, and other brain cells.

## 5 MICROGLIA-ASTROCYTE CROSSTALK REPAIRS POST-STROKE NEUROINFLAMMATION

The inhibition of inflammation between microglia and astrocytes can be achieved through cytokines, transcription factors, specific proteins, extracellular vesicles, and mitochondria.

### 5.1 Microglia Regulate Astrocytes

#### 5.1.1 IL-10/IL-10R

IL-10 is a key anti-inflammatory cytokine, produced not only by Th2 cells or Treg cells but also acts as a feedback modulator of various immune responses [140]. In IS, IL-10 is produced by regulatory T cells, macrophages, and microglia [141]. In ICH, microglia-derived IL-10 can trigger a series of intracellular signal transduction events, activating signal transducer and activator of transcription 3 (STAT3)-dependent pathway to accelerate hematoma clearance and inhibit the production of pro-inflammatory cytokines [142, 143]. Clinical trials have shown that IL-10 can serve as an independent predictor for stroke-related infections and may predict the prognosis in patients with acute IS [144, 145]. In IS, IL-10 not only controls the IL-17A-driven inflammatory response in peripheral immunity but also directly suppresses the production of IL-17A in γδ T cells in the ischemic brain [141]. The IL-10 receptor (mainly IL-10 Ra) is widely expressed in various cells. Studies have shown that IL-10R on the surface of astrocytes can bind to IL-10 produced by M2 microglia, stimulate astrocytes to secrete TGF-β, further increase the expression of microglia CXC3R1 and IL-4α, and attenuate microglia activation in the feedback loop, thus exerting anti-inflammatory effects [146]. Moreover, another study has found that IL-10 can alleviate lipid ROS accumulation and iron death in oligodendrocyte precursor cells (OPCs) after ICH, suggesting a novel mechanism of cell crosstalk under inflammatory conditions [147].

#### 5.1.2 IGF-1/IGF-1R

Insulin-like growth factor-1 (IGF-1) in the CNS is primarily derived from microglia and has been shown to promote neurotrophic effects and vascular regeneration after experimental IS and reduce brain injury. Astrocytes and neurons, typically acting as target cells, overexpress the IGF-1 receptor after brain injury, participating in intercellular interactions [148, 149]. IGF-1 receives signals from IGF-1R and exerts its growth and metabolic effects through the downstream PI3K/Akt pathway [150]. Recently, using spatial transcriptomics and transcriptome sequencing, researchers identified potential IGF-1/IGF-1R ligand-receptor pairs between microglia and astrocytes in a collagenase-induced ICH model. This interaction was particularly strong at day 7 after brain injury, concentrated in the core lesion area where the cells were located. Further studies revealed that early after ICH, IGF-1 derived from microglia induced neuroprotective scar formation in astrocytes through mTOR signaling activation [151]. Thus, IGF-1/IGF-1R/mTOR mediates microglia-astrocyte crosstalk and attenuates neuroinflammation.

#### 5.1.3 ZEB1

Zinc finger E-box binding homeobox-1 (ZEB1) is a member of the transcription factor family involved in epithelial-mesenchymal transition (EMT), driving cellular plasticity in tissues and regulating cell differentiation and specific cellular functions [152, 153]. ZEB1 plays a dual role in inducing and eliminating inflammation in microglia. After cerebral ischemia, ZEB1 is highly expressed in microglia and highly associated with its larger branched morphology, which favors the pro-inflammatory phenotype [154]. Conversely, ZEB1 induction promotes neuroprotection and cell survival in the neocortex after cerebral ischemia [155]. Studies have shown that ZEB1 limits inflammation by regulating macrophage metabolism, inhibiting mitochondrial translation and ROS production, and promoting macrophage transition to an immunosuppressive state. Recent research indicated that ZEB1 also acts as a medium in the interaction between microglia and astrocytes to attenuate neuroinflammation. In the tMCAO model, microglia-derived ZEB1 was upregulated in the ischemic penumbra, inhibiting astrocytic CXCL1 production through a TGF-β1-dependent pathway, thereby reducing neutrophil infiltration [156].

#### 5.1.4 Microglial-Derived Extracellular Vesicles

Extracellular vesicles (EVs) refer to double-layered membrane vesicles detached from the cell membrane or secreted by cells. According to their size and biogenesis, they are divided into exosomes, microvesicles, and apoptotic bodies [157]. EVs are a mechanism of intercellular communication, a carrier where cells exchange proteins, lipids, and genetic information [158]. In the CNS, the specific release of EVs is thought to signal brain injury. In tMCAO mice, miR-124 in M2 microglial small EVs reduced glial scar formation and astrocyte proliferation and activation through miR-124/STAT3 signaling. Simultaneously, it decreased Notch1 expression, increased Sox2 expression, participated in the transformation of astrocytes into neural progenitor cells, alleviated neuroinflammation after stroke, and promoted neurological function recovery [159]. Xin *et al.* [160] experimentally demonstrated that microglial-derived EVs after hypoxia attenuated focal ipsilateral reactive astrogliosis and aggregation of AQP4 on the plasma membrane of cortical astrocytes, and alleviated neuroinflammation in the peri-infarct cortex.

### 5.2 Astrocytes Induce M2 Microglia

#### 5.2.1 IL-33/ST2

IL-33 belongs to the IL-1 family. Once released upon cellular damage, it binds with the heterodimeric receptor ST2, participating in the immunomodulatory mechanism [161, 162]. CNS injuries can trigger damaged brain cells to release IL-33, primarily produced by oligodendrocytes and astrocytes [163, 164], while ST2 mainly colocalizes with microglia [165]. Under physiological conditions, IL-33 produced by astrocytes is involved in regulating the maturation and reconstruction of neural circuits by microglial cells to clear synapses and maintain synaptic homeostasis [166]. IL-33 is an important endogenous regulator in stroke, which can downregulate the level of pro-inflammatory cytokines, inhibit apoptosis and autophagy, and exert anti-inflammatory effects on brain edema caused by ICH [167]. In IS, activation of the IL-33/ST2 signaling pathway can cause an anti-inflammatory response in microglia, induce M2 microglial polarization, inhibit the expression of pro-inflammatory cytokines, and stimulate microglia to produce IL-10, thereby protecting ischemic neurons and reducing inflammatory responses. Furthermore, IL-4 released from neurons after stroke may synergistically regulate microglial responses with IL-33, providing neuroprotection [168, 169].

#### 5.2.2 ORM2

Orosomucoid (ORM) is located in the endoplasmic reticulum and is an acute phase response protein. It has various biological activities, such as involvement in protein quality control and co-regulation of lipid status, modulation of immunity, and maintenance of capillary barrier integrity [170, 171]. ORM reduces the production of pro-inflammatory cytokines in the ischemic penumbra, decreases malondialdehyde levels, significantly alleviates inflammation, improves BBB permeability, and shifts the balance from oxidative stress to antioxidant defense, thus preventing stroke [172]. ORM2 is the major subtype involved in IS and is believed to be protective. Jo *et al.* [173] found that in the LPS-induced neuroinflammation model, astrocytes are the major cellular source of ORM2 in the inflamed brain. ORM2 inhibits microglial activation and migration by blocking the interaction between CCL4 and CCR5, which is critical for its anti-inflammatory function.

#### 5.2.3 Astrocyte-Derived Extracellular Vesicles

After stroke, astrocyte-released extracellular vesicles (EVs) serve as vehicles for information transmission to establish communication with microglia. Liu *et al.* [174] found that in the oxygen-glucose deprivation (OGD) cell model, miR-29a in astrocyte-derived EVs downregulated TP53INP1 and inhibited NF-κB/NLRP3 pathway in microglia-related inflammatory response, reduced ischemia-reperfusion injury, and improved neurological function in rats after ischemia. Moreover, exosomes from astrocytes enriched with miR-873a-5p [175] and miR-148a-3p [176] could significantly inhibit LPS-induced microglial inflammation and promote M2 microglial polarization by reducing the phosphorylation of ERK and NF-κB p65, thus ameliorating neurological deficits.

#### 5.2.4 Mitochondrial Transfer

**Fig. (3):**
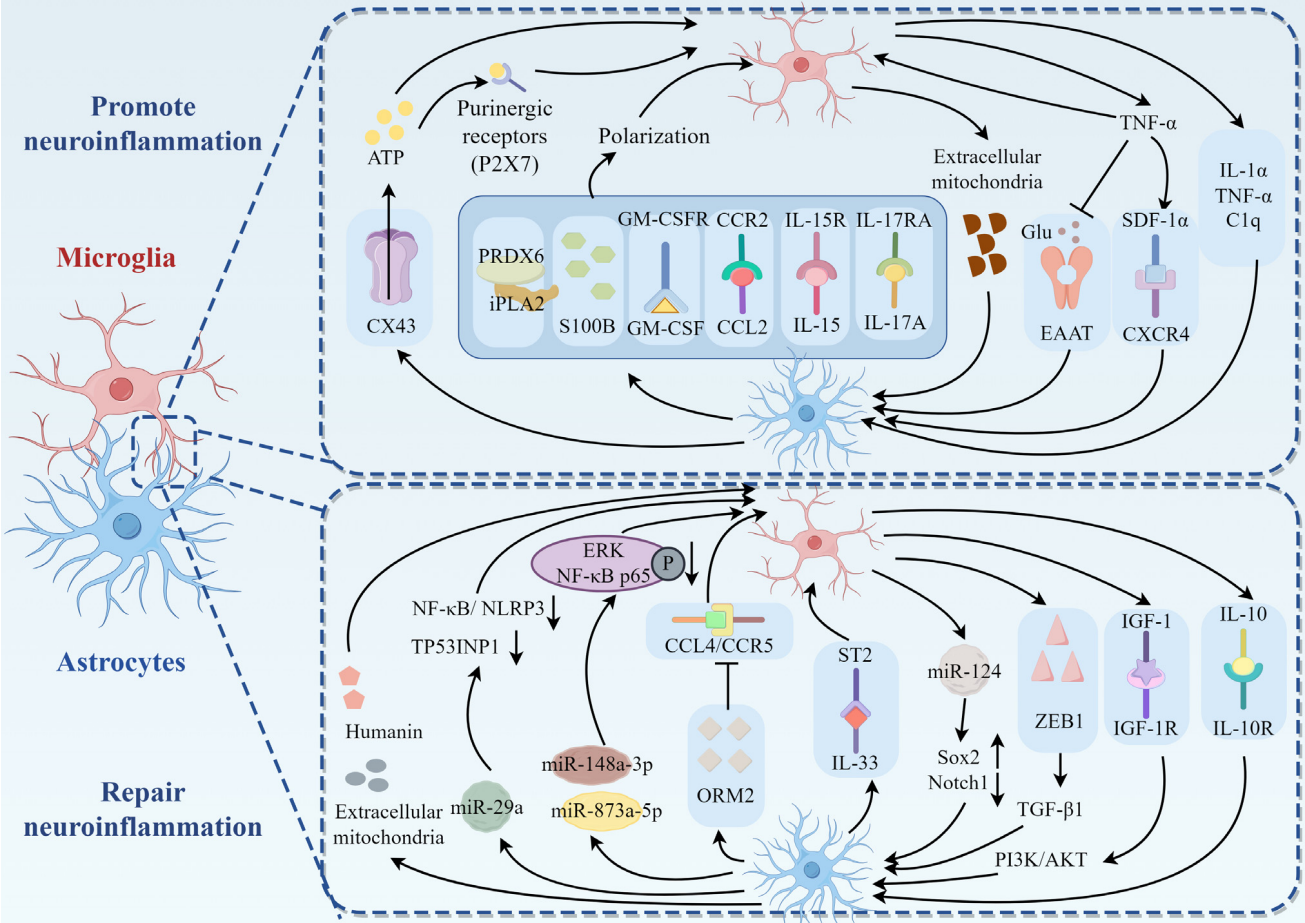
Pro-inflammatory and anti-inflammatory pathways related to stroke in microglia-astrocyte crosstalk. (Created by Figdraw).

The mechanism of mitochondrial exchange for glial crosstalk after stroke is gradually being elucidated. Early studies by Hayakawa *et al.* [177] found that astrocytes released extracellular mitochondrial particles after cerebral ischemia to adjacent neurons, promoted survival and plasticity after injury, which was mediated by a calcium-dependent mechanism involving CD38/cyclic ADP ribose signaling. Recently, Jung *et al.* [178, 179] discovered that after ICH, astrocytes released intact mitochondria and small bioactive peptide humanin (HN) into neurons, which restored antioxidant defense mediated by the neuronal mitochondrial enzyme manganese superoxide dismutase (Mn-SOD) and enhance neuronal plasticity. Mitochondria and HN released by astrocytes could also enter into microglia, promoting a “reparative” microglial phenotype, enhancing microglial phagocytosis, and reducing neuroinflammation. Thus, mitochondrial transfer has emerged as a novel mechanism in the interaction between microglia and astrocytes (Fig. **3**).

## 6 COMMUNICATION BETWEEN PERIPHERAL INFLAMMATORY CELLS AND MICROGLIA/ ASTROCYTES IN POST-STROKE NEUROINFLAMMATION

In addition to CNS glial cells, the infiltration of inflammatory cells plays a crucial role in the onset and progression of post-stroke neuroinflammation. Neutrophils are the first peripheral cells to infiltrate the lesion after stroke onset. They enhance neurotoxicity by releasing various mediators such as MMPs, ROS, IL-1β, collagenase, *etc*., and participate in microglia-astrocyte crosstalk [180]. Pro-inflammatory cytokines stimulate astrocytes to secrete more chemokines, leading to neutrophil migration, which depends on transforming growth factor beta-associated kinase 1 (TAK1) signaling [181]. Upregulation of transient receptor potential canonical 1 (TRPC1) in microglia stimulates the production of CCL5/2, resulting in increased neutrophil infiltration in the brain, thus aggravating neuroinflammation [182]. However, neutrophils are not merely harmful but may exhibit “functional plasticity” properties similar to macrophages, with a dual functional phenotype of pro-inflammatory (N1) and anti-inflammatory (N2) [183]. Recent evidence suggests that after stroke, microglia clear infiltrating neutrophils through phagocytosis, thereby alleviating neutrophil-mediated neurovascular destruction after brain injury [184, 185]. Inhibition of microglia by blocking colony-stimulating factor 1 receptor (CSF1R) significantly increased neutrophil infiltration in the ischemic core, exacerbating inflammatory responses [186]. Zhao *et al.* [187] found that microglia-derived IL-27 promoted the polarization of polymorphonuclear neutrophils to a less neurotoxic phenotype to limit injury.

After the stroke, astrocytes can promote the migration and infiltration of monocytes from circulation, spleen, and bone marrow into the brain parenchyma, mainly through the CCL2/CCR2 axis. This leads monocytes to differentiate into macrophages at the lesion while also stimulating the production of more M1 microglia, thus exacerbating inflammation [188]. However, monocytes/macrophages (MMs) can transform into an anti-inflammatory phenotype during the post-stroke recovery period, which depends on anti-inflammatory macrophages, particularly under the influence of IL-13, to alleviate neuroinflammation after IS [189, 190]. MMs secrete osteopontin (OPN) in ischemic tissues, inducing astrocytic processes to extend and cover the lesion core, reducing the continuous leakage of the BBB, and aiding in the resolution of edema [191].

Peripheral lymphocytes were stimulated, with an increase in CD4^+^/CD8^+^ T cells and B cells observed within 4 days after stroke [192]. After cerebral ischemia, microglia could stimulate activated CD4^+^ T cells to differentiate into Th1 or Th2 cells, which in turn produced pro-inflammatory or anti-inflammatory cytokines to modulate inflammation [193]. Microglia-derived chemokines CCL2/CCL8 promote cytotoxic CD8^+^ T cell infiltration and aggravate brain injury [194]. Treg cells express high levels of amphiregulin (Areg) and OPN, which are thought to inhibit IL-6 and STAT3 pathways in microglia and astrocytes after stroke and reduce inflammation [195]. However, microglia can, in turn, induce the expression of hypoxia-inducible factor 1-alpha (HIF-1α) in Treg cells through cell-to-cell contact, increasing Sirt2 expression in Treg cells and inhibiting their anti-inflammatory function [196]. Additionally, γδ T cells co-stimulated astrocytes to secrete CXCL1 through IL-17A and TNF-α, resulting in increased recruitment of neutrophils and exacerbation of inflammation [197].

## 7 THERAPEUTIC STRATEGIES

### 7.1 Targeting M2 Microglial Polarization

In the early stages of stroke, changes in signaling pathways after brain injury directly impact the type of microglia. Common signaling pathways involved in microglial polarization include AMPK, NF-κB, JAK/STAT, Notch, TLRs, and PPAR-γ. These pathways have been extensively studied and confirmed as therapeutic targets for stroke [198]. Baicalein and α-lipoic acid inhibit microglial NF-κB signaling by regulating the phosphorylation and nuclear translocation of p65, suppressing the expression of M1 markers, promoting M2 polarization, and reducing the release of pro-inflammatory cytokines such as IL-6, IL-18, and TNF-α [199, 200]. Moreover, MMPs and NLRP3 inflammasomes are key factors in regulating microglial polarization. Sinomenine can inhibit microglial caspase-3 activity, attenuate MMP3/9 expression, promote M2 polarization, and regulate neuroinflammation [201]. Mitoquinone promotes the transformation of microglia into the M2 phenotype after ICH by inhibiting the mitochondrial ROS/NLRP3 inflammasome pathway and increasing the high expression of CD36 on cells associated with hematoma absorption, thus improving neurological function [202].

### 7.2 Targeting Neuroprotective Astrocytes

After stroke, depending on time and environment, astrocytes may exacerbate injury or promote repair. Studies have shown that reduced astrocyte response is often associated with smaller infarct areas. Therefore, therapeutic strategies targeting astrocytes are mainly focused on inhibiting their activation, enhancing their anti-excitotoxicity, and promoting the activation of an anti-inflammatory phenotype. For example, inhibition of cycle-dependent kinases reduces astrocyte proliferation and neuronal death after MCAO [203]. TGN-020, an AQP4 inhibitor, reduces early brain edema, peri-infarct astrocyte proliferation, and infarct volume [204]. The free radical scavenger Edaravone reduces the number of astrocytes and microglia after propofo-induced brain injury through the BDNF/TrkB pathway and reverses the apoptosis and inflammatory response of nerve cells [205]. Ginsenoside Rb1 inhibits astrocyte activation after ischemia, promotes mitochondrial transfer, and supports neuronal survival [206]. Baicalin protects astrocytic Glu synthase protein from oxidative stress during cerebral ischemia-reperfusion injury, promotes Glu uptake, and resists excitotoxicity [207]. Supplementation of exogenous N-3 polyunsaturated fatty acids reduces pro-inflammatory astrocytes and alleviates neuroinflammation in cerebral ischemia/reperfusion stroke [208]. Kruppel-like transcription factor-4 (KLF-4), an evolutionarily conserved zinc finger transcription factor, is upregulated after cerebral ischemic injury and plays an anti-inflammatory role by regulating NF-κB to promote neuroprotective astrocyte polarization [58].

### 7.3 Acting on Microglia-Astrocyte Crosstalk

After stroke, microglia are the first to respond to inflammation and can induce amplification of inflammation. Then, could the depletion of microglia phenotype/function address the damage caused by intercellular crosstalk? Microglial survival depends on CSF1R signaling. After cerebral ischemia, depleting microglia with the inhibitor PLX3397 significantly increases inflammatory mediators produced by astrocytes, along with increased leukocyte infiltration and cell death, worsening brain infarction, and neurological deficits. PLX3397 itself does not alter the inflammatory state, indicating that the neuroprotective effect of microglia may stem from their role in suppressing astrocyte responses [209]. Similarly, the depletion of microglia increased neuroinflammation after administration of the inhibitors PLX5622 and AFS98 in the acute phase of cerebral ischemia in elderly mice. Although aged microglia are in a dysregulated state, they appear to have beneficial effects in the early stages of IS [210]. Li *et al.* [211] found that selective depletion of Arg1-positive microglia exacerbated ischemic injury by promoting inflammatory responses. However, Zeyen *et al.* [212] found that microglia-specific TAK1 depletion could attenuate post-ischemic neuroinflammation and apoptosis in the acute phase. After ICH, CSF1R inhibition effectively depleted microglia, seemingly affecting only the inflammatory state, improving neurological defects and cerebral edema [213]. Recent studies have shown that in the early stages of ICH, sustained microglial depletion leads to glial scar disorder and enhanced neutrophil infiltration. In the chronic stages, glial scars become destructive, at which time PLX3397 treatment provides significant benefits [151]. This suggests to us that, depending on the type of stroke and the complex temporal dynamics and overall net effects of microglia and astrocytes, it is crucial to deplete microglia at precise time points to regulate neuroinflammation effectively.

Current experiments have demonstrated significant therapeutic effects by acting on one type of cell and then indirectly on another. 10% hypertonic saline (HS) can inhibit the activation of microglial NLRP3 inflammasome and act on astrocytes through IL-1β/IL1R1/pNF-κB signaling pathway, down-regulating the expression of VEGF, reducing the permeability of the BBB, and alleviating inflammation and nerve damage after IS [214]. After ICH, crosstalk between astrocytic C3 and microglial C3aR regulates the C3-C3aR signaling pathway and inhibits microglial phagocytosis of myelin fragments. This process can be reversed by cerium nanoparticle (CeNP) treatment [215]. 2-carbonate-cyclophosphatidic acid (2ccPA), a more stable derivative of CPA, mediates communication between microglia and astrocytes and has neuroprotective effects. Astrocytes treated with 2ccPA reduce the secretion of C3 and regulate microglial differentiation to the M2 phenotype [216]. Glutathione S-transferase 1 (GSTM1) promotes a positive feedback pro-inflammatory loop between microglia and astrocytes during brain inflammation, and specific GST inhibitors may be used as therapeutic agents [217]. Comprehensive research on microglia-astrocyte crosstalk may contribute to innovative therapeutic approaches for stroke treatment and will likely become a focus for future drug development.

## CONCLUSION AND PERSPECTIVES

Post-stroke neuroinflammation is dynamic, with moderate inflammation exerting neuroprotective effects, helping to maintain the integrity of neuronal structure and function, promoting synaptic formation, and maintaining homeostasis within the CNS. Conversely, excessive inflammation is neurotoxic, leading to cell death and neural dysfunction, becoming a significant trigger or even a driving force for SBI.

The impact of microglia and astrocytes on neuroinflammation depends on distinct phenotypes and functions. Recent research on microglia-astrocyte crosstalk have provided unique insights into the role of the CNS in health and diseases. These cells engage in a molecular dialogue through secretion. Cytokines released by microglia can determine the function and fate of astrocytes. Astrocytes can influence the morphology, activation state, and function of microglia.

Therefore, we aim to further investigate and reveal the molecular mechanisms and pathways regulating neuroinflammation from a new perspective of microglia-astrocyte crosstalk, providing valuable insights into new therapeutic targets for stroke treatment. Additionally, the spatiotemporal dynamics of microglia and astrocytes may be crucial for understanding this crosstalk. Hence, therapeutic strategies should focus on the timing of microglial depletion and emphasize the transformation of microglia and astrocytes to a “protective” functional phenotype. With advancements in technology, the combined application of single-cell and spatial transcriptomics can more accurately and effectively identify different glial cell subtypes involved in interaction dialogues, infer potential mechanisms and target molecules of interactions between different cell types, and construct dynamic cellular maps for disease treatment, which have been successfully applied in research on various CNS diseases [218-220]. Apart from the interactions between microglia and astrocytes, do similar multidimensional crosstalk also exist among them and oligodendrocytes, neurons, and peripheral immune cells? This remains to be considered and studied, with the goal of fully utilizing the beneficial functions of glial cells, reducing inflammatory responses, and preventing secondary injury.

## LIST OF ABBREVIATIONS

2ccPA: 2-carbonate-cyclophosphatidic Acid
AA: Arundic Acid
AQP4: Aquaporin Protein-4
Areg: Amphiregulin
BBB: Blood-brain Barrier
C3: Complement C3
CCR2: Chemokine Ligand 2 Receptor
CeNP: Ceria Nanoparticle
CNS: Central Nervous System
CSF1R: Colony-stimulating Factor 1 Receptor
CSPGs: Chondroitin Sulfate Proteoglycans
Cx43: Connexin 43
DAMPs: Damage-associated Molecular Patterns
Drp1: Dynamin-associated Protein 1
EAAT: Excitatory Amino Acid Transporter
EMT: Epithelial-mesenchymal Transition
EVs: Extracellular Vesicles
GFAP: Glial Fibrillary Acidic Protein
Glu: Glutamate
GM-CSF: Granulocyte-macrophage Colony-stimulating Factor
HIF-1α: Hypoxia-inducible Factor 1-alpha
HMGB1: High Mobility Group Box-1 Protein
HN: Humanin
HS: Hemorrhagic Stroke
ICH: Intracerebral Hemorrhage
IGF-1: Insulin-like Growth Factor-1
IL: Interleukin
iPLA2: Independent Phospholipase A2
IS: Ischemic Stroke
LPS: Lipopolysaccharide
M-CSF: Macrophage Colony Stimulating Factor
MCP-1: Monocyte Chemotaxis Protein-1
MMPs: Matrix Metalloproteinases
MMs: Monocytes/Macrophages
Mn-SOD: Manganese Superoxide Dismutase
NK: Natural Killer
NLRs: NOD-like Receptors
OGD: Oxygen-glucose Deprivation
OPCs: Oligodendrocyte Precursor Cells
OPN: Osteopontin
ORM: Orosomucoid
PAR1: Protease-activated Receptor 1
pMCAO: Permanent Middle Cerebral Artery Occlusion
PRDX6: Peroxiredoxin 6
PRRs: Pattern Recognition Receptors
RNS: Reactive Nitrogen Species
ROS: Reactive Oxygen Species
SAH: Subarachnoid Hemorrhage
SAM: Stroke-associated Microglia
SBI: Secondary Brain Injury
STAT3: Signal Transducer and Activator of Transcription 3
TAK1: Transforming Growth Factor Beta-associated Kinase 1
TGF-β: Transforming Growth Factor-β
TLRs: Toll-like Receptors
tMCAO: Transient Middle Cerebral Artery Occlusion
TNF-α: Tumor Necrosis Factor-alpha
TRPC1: Transient Receptor Potential Canonical 1
ZEB1: Zinc Finger E-box Binding Homeobox-1

## References

[1] Feigin V.L., Stark B.A., Johnson C.O., Roth G.A., Bisignano C., Abady G.G., Abbasifard M., Abbasi-Kangevari M., Abd-Allah F., Abedi V., Abualhasan A., Abu-Rmeileh N.M.E., Abushouk A.I., Adebayo O.M., Agarwal G., Agasthi P., Ahinkorah B.O., Ahmad S., Ahmadi S., Ahmed Salih Y., Aji B., Akbarpour S., Akinyemi R.O., Al Hamad H., Alahdab F., Alif S.M., Alipour V., Aljunid S.M., Almustanyir S., Al-Raddadi R.M., Al-Shahi Salman R., Alvis-Guzman N., Ancuceanu R., Anderlini D., Anderson J.A., Ansar A., Antonazzo I.C., Arabloo J., Ärnlöv J., Artanti K.D., Aryan Z., Asgari S., Ashraf T., Athar M., Atreya A., Ausloos M., Baig A.A., Baltatu O.C., Banach M., Barboza M.A., Barker-Collo S.L., Bärnighausen T.W., Barone M.T.U., Basu S., Bazmandegan G., Beghi E., Beheshti M., Béjot Y., Bell A.W., Bennett D.A., Bensenor I.M., Bezabhe W.M., Bezabih Y.M., Bhagavathula A.S., Bhardwaj P., Bhattacharyya K., Bijani A., Bikbov B., Birhanu M.M., Boloor A., Bonny A., Brauer M., Brenner H., Bryazka D., Butt Z.A., Caetano dos Santos F.L., Campos-Nonato I.R., Cantu-Brito C., Carrero J.J., Castañeda-Orjuela C.A., Catapano A.L., Chakraborty P.A., Charan J., Choudhari S.G., Chowdhury E.K., Chu D-T., Chung S-C., Colozza D., Costa V.M., Costanzo S., Criqui M.H., Dadras O., Dagnew B., Dai X., Dalal K., Damasceno A.A.M., D’Amico E., Dandona L., Dandona R., Darega Gela J., Davletov K., De la Cruz-Góngora V., Desai R., Dhamnetiya D., Dharmaratne S.D., Dhimal M.L., Dhimal M., Diaz D., Dichgans M., Dokova K., Doshi R., Douiri A., Duncan B.B., Eftekharzadeh S., Ekholuenetale M., El Nahas N., Elgendy I.Y., Elhadi M., El-Jaafary S.I., Endres M., Endries A.Y., Erku D.A., Faraon E.J.A., Farooque U., Farzadfar F., Feroze A.H., Filip I., Fischer F., Flood D., Gad M.M., Gaidhane S., Ghanei Gheshlagh R., Ghashghaee A., Ghith N., Ghozali G., Ghozy S., Gialluisi A., Giampaoli S., Gilani S.A., Gill P.S., Gnedovskaya E.V., Golechha M., Goulart A.C., Guo Y., Gupta R., Gupta V.B., Gupta V.K., Gyanwali P., Hafezi-Nejad N., Hamidi S., Hanif A., Hankey G.J., Hargono A., Hashi A., Hassan T.S., Hassen H.Y., Havmoeller R.J., Hay S.I., Hayat K., Hegazy M.I., Herteliu C., Holla R., Hostiuc S., Househ M., Huang J., Humayun A., Hwang B-F., Iacoviello L., Iavicoli I., Ibitoye S.E., Ilesanmi O.S., Ilic I.M., Ilic M.D., Iqbal U., Irvani S.S.N., Islam S.M.S., Ismail N.E., Iso H., Isola G., Iwagami M., Jacob L., Jain V., Jang S-I., Jayapal S.K., Jayaram S., Jayawardena R., Jeemon P., Jha R.P., Johnson W.D., Jonas J.B., Joseph N., Jozwiak J.J., Jürisson M., Kalani R., Kalhor R., Kalkonde Y., Kamath A., Kamiab Z., Kanchan T., Kandel H., Karch A., Katoto P.D.M.C., Kayode G.A., Keshavarz P., Khader Y.S., Khan E.A., Khan I.A., Khan M., Khan M.A.B., Khatib M.N., Khubchandani J., Kim G.R., Kim M.S., Kim Y.J., Kisa A., Kisa S., Kivimäki M., Kolte D., Koolivand A., Koulmane Laxminarayana S.L., Koyanagi A., Krishan K., Krishnamoorthy V., Krishnamurthi R.V., Kumar G.A., Kusuma D., La Vecchia C., Lacey B., Lak H.M., Lallukka T., Lasrado S., Lavados P.M., Leonardi M., Li B., Li S., Lin H., Lin R-T., Liu X., Lo W.D., Lorkowski S., Lucchetti G., Lutzky Saute R., Magdy Abd El Razek H., Magnani F.G., Mahajan P.B., Majeed A., Makki A., Malekzadeh R., Malik A.A., Manafi N., Mansournia M.A., Mantovani L.G., Martini S., Mazzaglia G., Mehndiratta M.M., Menezes R.G., Meretoja A., Mersha A.G., Miao J.J., Miazgowski B., Miazgowski T., Michalek I.M., Mirrakhimov E.M., Mohammad Y., Mohammadian-Hafshejani A., Mohammed S., Mokdad A.H., Mokhayeri Y., Molokhia M., Moni M.A., Montasir A.A., Moradzadeh R., Morawska L., Morze J., Muruet W., Musa K.I., Nagarajan A.J., Naghavi M., Narasimha Swamy S., Nascimento B.R., Negoi R.I., Neupane Kandel S., Nguyen T.H., Norrving B., Noubiap J.J., Nwatah V.E., Oancea B., Odukoya O.O., Olagunju A.T., Orru H., Owolabi M.O., Padubidri J.R., Pana A., Parekh T., Park E-C., Pashazadeh Kan F., Pathak M., Peres M.F.P., Perianayagam A., Pham T-M., Piradov M.A., Podder V., Polinder S., Postma M.J., Pourshams A., Radfar A., Rafiei A., Raggi A., Rahim F., Rahimi-Movaghar V., Rahman M., Rahman M.A., Rahmani A.M., Rajai N., Ranasinghe P., Rao C.R., Rao S.J., Rathi P., Rawaf D.L., Rawaf S., Reitsma M.B., Renjith V., Renzaho A.M.N., Rezapour A., Rodriguez J.A.B., Roever L., Romoli M., Rynkiewicz A., Sacco S., Sadeghi M., Saeedi Moghaddam S., Sahebkar A., Saif-Ur-Rahman K.M., Salah R., Samaei M., Samy A.M., Santos I.S., Santric-Milicevic M.M., Sarrafzadegan N., Sathian B., Sattin D., Schiavolin S., Schlaich M.P., Schmidt M.I., Schutte A.E., Sepanlou S.G., Seylani A., Sha F., Shahabi S., Shaikh M.A., Shannawaz M., Shawon M.S.R., Sheikh A., Sheikhbahaei S., Shibuya K., Siabani S., Silva D.A.S., Singh J.A., Singh J.K., Skryabin V.Y., Skryabina A.A., Sobaih B.H., Stortecky S., Stranges S., Tadesse E.G., Tarigan I.U., Temsah M-H., Teuschl Y., Thrift A.G., Tonelli M., Tovani-Palone M.R., Tran B.X., Tripathi M., Tsegaye G.W., Ullah A., Unim B., Unnikrishnan B., Vakilian A., Valadan Tahbaz S., Vasankari T.J., Venketasubramanian N., Vervoort D., Vo B., Volovici V., Vosoughi K., Vu G.T., Vu L.G., Wafa H.A., Waheed Y., Wang Y., Wijeratne T., Winkler A.S., Wolfe C.D.A., Woodward M., Wu J.H., Wulf Hanson S., Xu X., Yadav L., Yadollahpour A., Yahyazadeh Jabbari S.H., Yamagishi K., Yatsuya H., Yonemoto N., Yu C., Yunusa I., Zaman M.S., Zaman S.B., Zamanian M., Zand R., Zandifar A., Zastrozhin M.S., Zastrozhina A., Zhang Y., Zhang Z-J., Zhong C., Zuniga Y.M.H., Murray C.J.L. (2021). Global, regional, and national burden of stroke and its risk factors, 1990–2019: A systematic analysis for the Global Burden of Disease Study 2019.. Lancet Neurol..

[2] Ma Q., Li R., Wang L., Yin P., Wang Y., Yan C., Ren Y., Qian Z., Vaughn M.G., McMillin S.E., Hay S.I., Naghavi M., Cai M., Wang C., Zhang Z., Zhou M., Lin H., Yang Y. (2021). Temporal trend and attributable risk factors of stroke burden in China, 1990–2019: An analysis for the global burden of disease study 2019.. Lancet Public Health.

[3] Shi K., Tian D.C., Li Z.G., Ducruet A.F., Lawton M.T., Shi F.D. (2019). Global brain inflammation in stroke.. Lancet Neurol..

[4] Tschoe C., Bushnell C.D., Duncan P.W., Alexander-Miller M.A., Wolfe S.Q. (2020). Neuroinflammation after intracerebral hemorrhage and potential therapeutic targets.. J. Stroke.

[5] Jayaraj R.L., Azimullah S., Beiram R., Jalal F.Y., Rosenberg G.A. (2019). Neuroinflammation: Friend and foe for ischemic stroke.. J. Neuroinflammation.

[6] Murao A., Aziz M., Wang H., Brenner M., Wang P. (2021). Release mechanisms of major DAMPs.. Apoptosis.

[7] Maida C.D., Norrito R.L., Daidone M., Tuttolomondo A., Pinto A. (2020). Neuroinflammatory mechanisms in ischemic stroke: Focus on cardioembolic stroke, background, and therapeutic approaches.. Int. J. Mol. Sci..

[8] Alsbrook D.L., Di Napoli M., Bhatia K., Biller J., Andalib S., Hinduja A., Rodrigues R., Rodriguez M., Sabbagh S.Y., Selim M., Farahabadi M.H., Jafarli A., Divani A.A. (2023). Neuroinflammation in acute ischemic and hemorrhagic stroke.. Curr. Neurol. Neurosci. Rep..

[9] Gong T., Liu L., Jiang W., Zhou R. (2020). DAMP-sensing receptors in sterile inflammation and inflammatory diseases.. Nat. Rev. Immunol..

[10] Keep R.F., Hua Y., Xi G. (2012). Intracerebral haemorrhage: Mechanisms of injury and therapeutic targets.. Lancet Neurol..

[11] Jin R., Liu L., Zhang S., Nanda A., Li G. (2013). Role of inflammation and its mediators in acute ischemic stroke.. J. Cardiovasc. Transl. Res..

[12] Sun M., You H., Hu X., Luo Y., Zhang Z., Song Y., An J., Lu H. (2023). Microglia–astrocyte interaction in neural development and neural pathogenesis.. Cells.

[13] Lopez-Ortiz A.O., Eyo U.B. (2024). Astrocytes and microglia in the coordination of CNS development and homeostasis.. J. Neurochem..

[14] Streit W., Xue Q.S. (2013). Microglial senescence.. CNS Neurol. Disord. Drug Targets.

[15] Du Y., Brennan F.H., Popovich P.G., Zhou M. (2022). Microglia maintain the normal structure and function of the hippocampal astrocyte network.. Glia.

[16] Vainchtein I.D., Chin G., Cho F.S., Kelley K.W., Miller J.G., Chien E.C., Liddelow S.A., Nguyen P.T., Nakao-Inoue H., Dorman L.C., Akil O., Joshita S., Barres B.A., Paz J.T., Molofsky A.B., Molofsky A.V. (2018). Astrocyte-derived interleukin-33 promotes microglial synapse engulfment and neural circuit development.. Science.

[17] Kirkley K.S., Popichak K.A., Afzali M.F., Legare M.E., Tjalkens R.B. (2017). Microglia amplify inflammatory activation of astrocytes in manganese neurotoxicity.. J. Neuroinflammation.

[18] Bhusal A., Afridi R., Lee W.H., Suk K. (2023). Bidirectional communication between microglia and astrocytes in neuroinflammation.. Curr. Neuropharmacol..

[19] Ginhoux F., Greter M., Leboeuf M., Nandi S., See P., Gokhan S., Mehler M.F., Conway S.J., Ng L.G., Stanley E.R., Samokhvalov I.M., Merad M. (2010). Fate mapping analysis reveals that adult microglia derive from primitive macrophages.. Science.

[20] Garaschuk O., Verkhratsky A. (2019). Physiology of microglia.. Methods Mol. Biol..

[21] Nayak D., Roth T.L., McGavern D.B. (2014). Microglia development and function.. Annu. Rev. Immunol..

[22] Franco R., Fernández-Suárez D. (2015). Alternatively activated microglia and macrophages in the central nervous system.. Prog. Neurobiol..

[23] Lacey D.C., Achuthan A., Fleetwood A.J., Dinh H., Roiniotis J., Scholz G.M., Chang M.W., Beckman S.K., Cook A.D., Hamilton J.A. (2012). Defining GM-CSF- and macrophage-CSF-dependent macrophage responses by in vitro models.. J. Immunol..

[24] Zhao H., Garton T., Keep R.F., Hua Y., Xi G. (2015). Microglia/macrophage polarization after experimental intracerebral hemorrhage.. Transl. Stroke Res..

[25] Hu X., Leak R.K., Shi Y., Suenaga J., Gao Y., Zheng P., Chen J. (2015). Microglial and macrophage polarization—new prospects for brain repair.. Nat. Rev. Neurol..

[26] Liu F., Cheng X., Zhao C., Zhang X., Liu C., Zhong S., Liu Z., Lin X., Qiu W., Zhang X. (2024). Single-cell mapping of brain myeloid cell subsets reveals key transcriptomic changes favoring neuroplasticity after ischemic stroke.. Neurosci. Bull..

[27] Kim S., Lee W., Jo H., Sonn S.K., Jeong S.J., Seo S., Suh J., Jin J., Kweon H.Y., Kim T.K., Moon S.H., Jeon S., Kim J.W., Kim Y.R., Lee E.W., Shin H.K., Park S.H., Oh G.T. (2022). The antioxidant enzyme Peroxiredoxin-1 controls stroke-associated microglia against acute ischemic stroke.. Redox Biol..

[28] Lai A.Y., Todd K.G. (2006). Microglia in cerebral ischemia: Molecular actions and interactions.. Can. J. Physiol. Pharmacol..

[29] Nakajima K., Kohsaka S. (2004). Microglia: Neuroprotective and neurotrophic cells in the central nervous system.. Curr. Drug Targets Cardiovasc. Haematol. Disord..

[30] Rupalla K., Allegrini P.R., Sauer D., Wiessner C. (1998). Time course of microglia activation and apoptosis in various brain regions after permanent focal cerebral ischemia in mice.. Acta Neuropathol..

[31] Ito D., Tanaka K., Suzuki S., Dembo T., Fukuuchi Y. (2001). Enhanced expression of Iba1, ionized calcium-binding adapter molecule 1, after transient focal cerebral ischemia in rat brain.. Stroke.

[32] Perego C., Fumagalli S., De Simoni M.G. (2011). Temporal pattern of expression and colocalization of microglia/macrophage phenotype markers following brain ischemic injury in mice.. J. Neuroinflammation.

[33] Zhu J., Cao D., Guo C., Liu M., Tao Y., Zhou J., Wang F., Zhao Y., Wei J., Zhang Y., Fang W., Li Y. (2019). Berberine facilitates angiogenesis against ischemic stroke through modulating microglial polarization via AMPK signaling.. Cell. Mol. Neurobiol..

[34] Hu X., Li P., Guo Y., Wang H., Leak R.K., Chen S., Gao Y., Chen J. (2012). Microglia/macrophage polarization dynamics reveal novel mechanism of injury expansion after focal cerebral ischemia.. Stroke.

[35] Shu Z.M., Shu X.D., Li H.Q., Sun Y., Shan H., Sun X.Y., Du R.H., Lu M., Xiao M., Ding J.H., Hu G., Ginkgolide B. (2016). Ginkgolide B protects against ischemic stroke via modulating microglia polarization in mice.. CNS Neurosci. Ther..

[36] Wang J., Doré S. (2007). Heme oxygenase-1 exacerbates early brain injury after intracerebral haemorrhage.. Brain.

[37] Xue M., Del Bigio M.R. (2000). Intracerebral injection of autologous whole blood in rats: Time course of inflammation and cell death.. Neurosci. Lett..

[38] Zhao X., Sun G., Zhang J., Strong R., Song W., Gonzales N., Grotta J.C., Aronowski J. (2007). Hematoma resolution as a target for intracerebral hemorrhage treatment: Role for peroxisome proliferator‐activated receptor γ in microglia/macrophages.. Ann. Neurol..

[39] Wei J., Wang M., Jing C., Keep R.F., Hua Y., Xi G. (2020). Multinucleated giant cells in experimental intracerebral hemorrhage.. Transl. Stroke Res..

[40] Yang S.S., Lin L., Liu Y., Wang J., Chu J., Zhang T., Ning L.N., Shi Y., Fang Y.Y., Zeng P., Wang J.Z., Qiu M.Y., Tian Q. (2016). High morphologic plasticity of microglia/macrophages following experimental intracerebral hemorrhage in rats.. Int. J. Mol. Sci..

[41] Wan S., Cheng Y., Jin H., Guo D., Hua Y., Keep R.F., Xi G. (2016). Microglia activation and polarization after intracerebral hemorrhage in mice: The role of protease-activated receptor-1.. Transl. Stroke Res..

[42] Lan X., Han X., Li Q., Li Q., Gao Y., Cheng T., Wan J., Zhu W., Wang J. (2017). Pinocembrin protects hemorrhagic brain primarily by inhibiting toll-like receptor 4 and reducing M1 phenotype microglia.. Brain Behav. Immun..

[43] Zheng Z.V., Lyu H., Lam S.Y.E., Lam P.K., Poon W.S., Wong G.K.C. (2020). The dynamics of microglial polarization reveal the resident neuroinflammatory responses after subarachnoid hemorrhage.. Transl. Stroke Res..

[44] Gris T., Laplante P., Thebault P., Cayrol R., Najjar A., Joannette-Pilon B., Brillant-Marquis F., Magro E., English S.W., Lapointe R., Bojanowski M., Francoeur C.L., Cailhier J.F. (2019). Innate immunity activation in the early brain injury period following subarachnoid hemorrhage.. J. Neuroinflammation.

[45] Bodhankar S., Lapato A., Chen Y., Vandenbark A.A., Saugstad J.A., Offner H. (2015). Role for microglia in sex differences after ischemic stroke: importance of M2.. Metab. Brain Dis..

[46] Suenaga J., Hu X., Pu H., Shi Y., Hassan S.H., Xu M., Leak R.K., Stetler R.A., Gao Y., Chen J. (2015). White matter injury and microglia/macrophage polarization are strongly linked with age-related long-term deficits in neurological function after stroke.. Exp. Neurol..

[47] Sofroniew M.V. (2020). Astrocyte reactivity: Subtypes, states, and functions in CNS Innate immunity.. Trends Immunol..

[48] Liu L., Liu J., Bao J., Bai Q., Wang G. (2020). Interaction of microglia and astrocytes in the neurovascular unit.. Front. Immunol..

[49] Liu Z., Chopp M. (2016). Astrocytes, therapeutic targets for neuroprotection and neurorestoration in ischemic stroke.. Prog. Neurobiol..

[50] Pekny M., Wilhelmsson U., Tatlisumak T., Pekna M. (2019). Astrocyte activation and reactive gliosis—A new target in stroke?. Neurosci. Lett..

[51] Luger S., Witsch J., Dietz A., Hamann G.F., Minnerup J., Schneider H., Sitzer M., Wartenberg K.E., Niessner M., Foerch C. (2017). Glial fibrillary acidic protein serum levels distinguish between intracerebral hemorrhage and cerebral ischemia in the early phase of stroke.. Clin. Chem..

[52] Kitchen P., Salman M.M., Halsey A.M., Clarke-Bland C., MacDonald J.A., Ishida H., Vogel H.J., Almutiri S., Logan A., Kreida S., Al-Jubair T., Winkel M.J., Gourdon P., Törnroth-Horsefield S., Conner M.T., Ahmed Z., Conner A.C., Bill R.M. (2020). Targeting aquaporin-4 subcellular localization to treat central nervous system edema.. Cell.

[53] Scimemi A. (2018). Astrocytes and the warning signs of intracerebral hemorrhagic stroke.. Neural Plast..

[54] Liddelow S.A., Guttenplan K.A., Clarke L.E., Bennett F.C., Bohlen C.J., Schirmer L., Bennett M.L., Münch A.E., Chung W.S., Peterson T.C., Wilton D.K., Frouin A., Napier B.A., Panicker N., Kumar M., Buckwalter M.S., Rowitch D.H., Dawson V.L., Dawson T.M., Stevens B., Barres B.A. (2017). Neurotoxic reactive astrocytes are induced by activated microglia.. Nature.

[55] Livne-Bar I., Wei J., Liu H.H., Alqawlaq S., Won G.J., Tuccitto A., Gronert K., Flanagan J.G., Sivak J.M. (2017). Astrocyte-derived lipoxins A4 and B4 promote neuroprotection from acute and chronic injury.. J. Clin. Invest..

[56] Yu G., Zhang Y., Ning B. (2021). Reactive astrocytes in central nervous system injury: Subgroup and potential therapy.. Front. Cell. Neurosci..

[57] Zhang Q., Liu C., Shi R., Zhou S., Shan H., Deng L., Chen T., Guo Y., Zhang Z., Yang G.Y., Wang Y., Tang Y. (2022). Blocking C3d+/GFAP+ A1 astrocyte conversion with semaglutide attenuates blood-brain barrier disruption in mice after ischemic stroke.. Aging Dis..

[58] Wang C., Li L. (2023). The critical role of KLF4 in regulating the activation of A1/A2 reactive astrocytes following ischemic stroke.. J. Neuroinflammation.

[59] Rakers C., Schleif M., Blank N., Matušková H., Ulas T., Händler K., Torres S.V., Schumacher T., Tai K., Schultze J.L., Jackson W.S., Petzold G.C. (2019). Stroke target identification guided by astrocyte transcriptome analysis.. Glia.

[60] Fei X., Dou Y., Wang L., Wu X., Huan Y., Wu S., He X., Lv W., Wei J., Fei Z. (2022). Homer1 promotes the conversion of A1 astrocytes to A2 astrocytes and improves the recovery of transgenic mice after intracerebral hemorrhage.. J. Neuroinflammation.

[61] Zhang L., Guo K., Zhou J., Zhang X., Yin S., Peng J., Liao Y., Jiang Y. (2021). Ponesimod protects against neuronal death by suppressing the activation of A1 astrocytes in early brain injury after experimental subarachnoid hemorrhage.. J. Neurochem..

[62] Ma M., Li H., Wu J., Zhang Y., Shen H., Li X., Wang Z., Chen G. (2020). Roles of prokineticin 2 in subarachnoid hemorrhage-induced early brain injury via regulation of phenotype polarization in astrocytes.. Mol. Neurobiol..

[63] Patabendige A., Singh A., Jenkins S., Sen J., Chen R. (2021). Astrocyte activation in neurovascular damage and repair following ischaemic stroke.. Int. J. Mol. Sci..

[64] Anderson M.A., Burda J.E., Ren Y., Ao Y., O’Shea T.M., Kawaguchi R., Coppola G., Khakh B.S., Deming T.J., Sofroniew M.V. (2016). Astrocyte scar formation aids central nervous system axon regeneration.. Nature.

[65] Gris P., Tighe A., Levin D., Sharma R., Brown A. (2007). Transcriptional regulation of scar gene expression in primary astrocytes.. Glia.

[66] Chen S.H., Oyarzabal E.A., Sung Y.F., Chu C.H., Wang Q., Chen S.L., Lu R.B., Hong J.S. (2015). Microglial regulation of immunological and neuroprotective functions of astroglia.. Glia.

[67] Liddelow S.A., Barres B.A. (2017). Reactive astrocytes: Production, function, and therapeutic potential.. Immunity.

[68] Huang Y., Chen S., Luo Y., Han Z. (2020). Crosstalk between inflammation and the BBB in stroke.. Curr. Neuropharmacol..

[69] Dejanovic B., Wu T., Tsai M.C., Graykowski D., Gandham V.D., Rose C.M., Bakalarski C.E., Ngu H., Wang Y., Pandey S., Rezzonico M.G., Friedman B.A., Edmonds R., De Mazière A., Rakosi-Schmidt R., Singh T., Klumperman J., Foreman O., Chang M.C., Xie L., Sheng M., Hanson J.E. (2022). Complement C1q-dependent excitatory and inhibitory synapse elimination by astrocytes and microglia in Alzheimer’s disease mouse models.. Nat. Aging.

[70] Tang S., Hu W., Zou H., Luo Q., Deng W., Cao S. (2024). The complement system: A potential target for the comorbidity of chronic pain and depression.. Korean J. Pain.

[71] Iram T., Ramirez-Ortiz Z., Byrne M.H., Coleman U.A., Kingery N.D., Means T.K., Frenkel D., El Khoury J. (2016). Megf10 is a receptor for C1Q that mediates clearance of apoptotic cells by astrocytes.. J. Neurosci..

[72] Färber K., Cheung G., Mitchell D., Wallis R., Weihe E., Schwaeble W., Kettenmann H. (2009). C1q, the recognition subcomponent of the classical pathway of complement, drives microglial activation.. J. Neurosci. Res..

[73] Zhang W., Ding L., Chen H., Zhang M., Ma R., Zheng S., Gong J., Zhang Z., Xu H., Xu P., Zhang Y. (2023). Cntnap4 partial deficiency exacerbates α-synuclein pathology through astrocyte–microglia C3-C3aR pathway.. Cell Death Dis..

[74] Korimerla N., Wahl D.R. (2021). A complementary strategy to mitigate radiation-induced cognitive decline.. Cancer Res..

[75] Hanisch U.K. (2002). Microglia as a source and target of cytokines.. Glia.

[76] Wagner K.R., Beiler S., Beiler C., Kirkman J., Casey K., Robinson T., Larnard D., de Courten-Myers G.M., Linke M.J., Zuccarello M. (2006). Delayed profound local brain hypothermia markedly reduces interleukin-1β gene expression and vasogenic edema development in a porcine model of intracerebral hemorrhage.. Acta Neurochir. Suppl..

[77] Clausen B.H., Lambertsen K.L., Babcock A.A., Holm T.H., Dagnaes-Hansen F., Finsen B. (2008). Interleukin-1beta and tumor necrosis factor-alpha are expressed by different subsets of microglia and macrophages after ischemic stroke in mice.. J. Neuroinflammation.

[78] Fernandes A., Barateiro A., Falcão A.S., Silva S.L.A., Vaz A.R., Brito M.A., Marques Silva R.F., Brites D. (2011). Astrocyte reactivity to unconjugated bilirubin requires TNF‐α and IL‐1β receptor signaling pathways.. Glia.

[79] Giulian D., Young D.G., Woodward J., Brown D.C., Lachman L.B. (1988). Interleukin-1 is an astroglial growth factor in the developing brain.. J. Neurosci..

[80] Pickering M., Cumiskey D., O’Connor J.J. (2005). Actions of TNF‐α on glutamatergic synaptic transmission in the central nervous system.. Exp. Physiol..

[81] Viviani B., Boraso M., Marchetti N., Marinovich M. (2014). Perspectives on neuroinflammation and excitotoxicity: A neurotoxic conspiracy?. Neurotoxicology.

[82] Jiang G., Li X., Liu M., Li H., Shen H. (2023). liao, J.; You, W.; Fang, Q.; Chen, G. Remote ischemic postconditioning ameliorates stroke injury via the SDF-1α/CXCR4 signaling axis in rats.. Brain Res. Bull..

[83] Chiazza F., Tammen H., Pintana H., Lietzau G., Collino M., Nyström T., Klein T., Darsalia V., Patrone C. (2018). The effect of DPP-4 inhibition to improve functional outcome after stroke is mediated by the SDF-1α/CXCR4 pathway.. Cardiovasc. Diabetol..

[84] Bonavia R., Bajetto A., Barbero S., Pirani P., Florio T., Schettini G. (2003). Chemokines and their receptors in the CNS: Expression of CXCL12/SDF-1 and CXCR4 and their role in astrocyte proliferation.. Toxicol. Lett..

[85] Hickey K.N., Grassi S.M., Caplan M.R., Stabenfeldt S.E. (2021). Stromal cell-derived factor-1a autocrine/paracrine signaling contributes to spatiotemporal gradients in the brain.. Cell. Mol. Bioeng..

[86] Bezzi P., Domercq M., Brambilla L., Galli R., Schols D., De Clercq E., Vescovi A., Bagetta G., Kollias G., Meldolesi J., Volterra A. (2001). CXCR4-activated astrocyte glutamate release via TNFα: Amplification by microglia triggers neurotoxicity.. Nat. Neurosci..

[87] Yang F., Luo W.J., Sun W., Wang Y., Wang J.L., Yang F., Li C.L., Wei N., Wang X.L., Guan S.M., Chen J. (2017). SDF1-CXCR4 signaling maintains central post-stroke pain through mediation of glial-neuronal interactions.. Front. Mol. Neurosci..

[88] Holm T.H., Draeby D., Owens T. (2012). Microglia are required for astroglial toll‐like receptor 4 response and for optimal TLR2 and TLR3 response.. Glia.

[89] van der Bliek A.M., Shen Q., Kawajiri S. (2013). Mechanisms of mitochondrial fission and fusion.. Cold Spring Harb. Perspect. Biol..

[90] He M., Wang X., Liu Z., Cui Q., Chen Y., Geng W., Zhu J., Shen J. (2022). CDK5 mediates proinflammatory effects of microglia through activated DRP1 phosphorylation in rat model of intracerebral hemorrhage.. Dis. Markers.

[91] Herst P.M., Rowe M.R., Carson G.M., Berridge M.V. (2017). Functional mitochondria in health and disease.. Front. Endocrinol..

[92] Liaudanskaya V., Fiore N.J., Zhang Y., Milton Y., Kelly M.F., Coe M., Barreiro A., Rose V.K., Shapiro M.R., Mullis A.S., Shevzov-Zebrun A., Blurton-Jones M., Whalen M.J., Symes A.J., Georgakoudi I., Nieland T.J.F., Kaplan D.L. (2023). Mitochondria dysregulation contributes to secondary neurodegeneration progression post-contusion injury in human 3D in vitro triculture brain tissue model.. Cell Death Dis..

[93] Joshi A.U., Minhas P.S., Liddelow S.A., Haileselassie B., Andreasson K.I., Dorn G.W., Mochly-Rosen D. (2019). Fragmented mitochondria released from microglia trigger A1 astrocytic response and propagate inflammatory neurodegeneration.. Nat. Neurosci..

[94] Liu W., Qi Z., Li W., Liang J., Zhao L., Shi Y. (2022). M1 microglia induced neuronal injury on ischemic stroke via mitochondrial crosstalk between microglia and neurons.. Oxid. Med. Cell. Longev..

[95] Lin Y., Zhang J-C., Yao C-Y., Wu Y., Abdelgawad A.F., Yao S-L., Yuan S-Y. (2016). Critical role of astrocytic interleukin-17 A in post-stroke survival and neuronal differentiation of neural precursor cells in adult mice.. Cell Death Dis..

[96] Dai Q., Li S., Liu T., Zheng J., Han S., Qu A., Li J. (2019). Interleukin‐17A‐mediated alleviation of cortical astrocyte ischemic injuries affected the neurological outcome of mice with ischemic stroke.. J. Cell. Biochem..

[97] Yu A., Duan H., Zhang T., Pan Y., Kou Z., Zhang X., Lu Y., Wang S., Yang Z. (2016). IL-17A promotes microglial activation and neuroinflammation in mouse models of intracerebral haemorrhage.. Mol. Immunol..

[98] Elain G., Jeanneau K., Rutkowska A., Mir A.K., Dev K.K. (2014). The selective anti-IL17A monoclonal antibody secukinumab (AIN457) attenuates IL17A-induced levels of IL6 in human astrocytes.. Glia.

[99] Chen X., Zhang Y., Ding Q., He Y., Li H. (2023). Role of IL-17A in different stages of ischemic stroke.. Int. Immunopharmacol..

[100] Liu G., Guo J., Liu J., Wang Z., Liang D. (2014). Toll-like receptor signaling directly increases functional IL-17RA expression in neuroglial cells.. Clin. Immunol..

[101] Li S., Dai Q., Yu J., Liu T., Liu S., Ma L., Zhang Y., Han S., Li J. (2017). Identification of IL-17A-derived neural cell type and dynamic changes of IL-17A in serum/CSF of mice with ischemic stroke.. Neurol. Res..

[102] Ma L., Pan X., Zhou F., Liu K., Wang L. (2018). Hyperforin protects against acute cerebral ischemic injury through inhibition of interleukin-17A-mediated microglial activation.. Brain Res..

[103] Li M., Li Z., Yao Y., Jin W.N., Wood K., Liu Q., Shi F.D., Hao J. (2017). Astrocyte-derived interleukin-15 exacerbates ischemic brain injury via propagation of cellular immunity.. Proc. Natl. Acad. Sci. USA.

[104] Gómez-Nicola D., Valle-Argos B., Pita-Thomas D.W., Nieto-Sampedro M. (2008). Interleukin 15 expression in the CNS: Blockade of its activity prevents glial activation after an inflammatory injury.. Glia.

[105] Lee G.A., Lin T.N., Chen C.Y., Mau S.Y., Huang W.Z., Kao Y.C., Ma R., Liao N.S. (2018). Interleukin 15 blockade protects the brain from cerebral ischemia-reperfusion injury.. Brain Behav. Immun..

[106] Perera L.P., Goldman C.K., Waldmann T.A. (1999). IL-15 induces the expression of chemokines and their receptors in T lymphocytes.. J. Immunol..

[107] Budagian V., Bulanova E., Paus R., Bulfonepaus S. (2006). IL-15/IL-15 receptor biology: A guided tour through an expanding universe.. Cytokine Growth Factor Rev..

[108] Stoklasek T.A., Schluns K.S., Lefrançois L. (2006). Combined IL-15/IL-15Ralpha immunotherapy maximizes IL-15 activity in vivo.. J. Immunol..

[109] Lee Y.B., Nagai A., Kim S.U. (2002). Cytokines, chemokines, and cytokine receptors in human microglia.. J. Neurosci. Res..

[110] Shi S.X., Li Y.J., Shi K., Wood K., Ducruet A.F., Liu Q. (2020). IL (Interleukin)-15 bridges astrocyte-microglia crosstalk and exacerbates brain injury following intracerebral hemorrhage.. Stroke.

[111] Mayo L., Trauger S.A., Blain M., Nadeau M., Patel B., Alvarez J.I., Mascanfroni I.D., Yeste A., Kivisäkk P., Kallas K., Ellezam B., Bakshi R., Prat A., Antel J.P., Weiner H.L., Quintana F.J. (2014). Regulation of astrocyte activation by glycolipids drives chronic CNS inflammation.. Nat. Med..

[112] Kooij G., Mizee M.R., van Horssen J., Reijerkerk A., Witte M.E., Drexhage J.A.R., van der Pol S.M.A., van het Hof B., Scheffer G., Scheper R., Dijkstra C.D., van der Valk P., de Vries H.E. (2011). Adenosine triphosphate-binding cassette transporters mediate chemokine (C-C motif) ligand 2 secretion from reactive astrocytes: Relevance to multiple sclerosis pathogenesis.. Brain.

[113] Tsukuda K., Mogi M., Iwanami J., Min L.J., Jing F., Oshima K., Horiuchi M. (2011). Irbesartan attenuates ischemic brain damage by inhibition of MCP-1/CCR2 signaling pathway beyond AT1 receptor blockade.. Biochem. Biophys. Res. Commun..

[114] Yao Y., Tsirka S.E. (2012). The CCL2‐CCR2 system affects the progression and clearance of intracerebral hemorrhage.. Glia.

[115] Zhang J., Shi X.Q., Echeverry S., Mogil J.S., De Koninck Y., Rivest S. (2007). Expression of CCR2 in both resident and bone marrow-derived microglia plays a critical role in neuropathic pain.. J. Neurosci..

[116] He M., Dong H., Huang Y., Lu S., Zhang S., Qian Y., Jin W. (2016). Astrocyte-derived CCL2 is associated with M1 activation and recruitment of cultured microglial cells.. Cell. Physiol. Biochem..

[117] Wheeler M.A., Clark I.C., Tjon E.C., Li Z., Zandee S.E.J., Couturier C.P., Watson B.R., Scalisi G., Alkwai S., Rothhammer V., Rotem A., Heyman J.A., Thaploo S., Sanmarco L.M., Ragoussis J., Weitz D.A., Petrecca K., Moffitt J.R., Becher B., Antel J.P., Prat A., Quintana F.J. (2020). MAFG-driven astrocytes promote CNS inflammation.. Nature.

[118] Parajuli B., Sonobe Y., Kawanokuchi J., Doi Y., Noda M., Takeuchi H., Mizuno T., Suzumura A. (2012). GM-CSF increases LPS-induced production of proinflammatory mediators via upregulation of TLR4 and CD14 in murine microglia.. J. Neuroinflammation.

[119] McLay R., Kimura M., Banks W.A., Kastin A.J. (1997). Granulocyte-macrophage colony-stimulating factor crosses the blood-- brain and blood-spinal cord barriers.. Brain.

[120] Re F., Belyanskaya S.L., Riese R.J., Cipriani B., Fischer F.R., Granucci F., Ricciardi-Castagnoli P., Brosnan C., Stern L.J., Strominger J.L., Santambrogio L. (2002). Granulocyte-macrophage colony-stimulating factor induces an expression program in neonatal microglia that primes them for antigen presentation.. J. Immunol..

[121] Kim S., Son Y. (2021). Astrocytes stimulate microglial proliferation and m2 polarization in vitro through crosstalk between astrocytes and microglia.. Int. J. Mol. Sci..

[122] Gonzalez L.L., Garrie K., Turner M.D. (2020). Role of S100 proteins in health and disease.. Biochim. Biophys. Acta Mol. Cell Res..

[123] Kabadi S.V., Stoica B.A., Zimmer D.B., Afanador L., Duffy K.B., Loane D.J., Faden A.I. (2015). S100B inhibition reduces behavioral and pathologic changes in experimental traumatic brain injury.. J. Cereb. Blood Flow Metab..

[124] Xu J., Wang H., Won S.J., Basu J., Kapfhamer D., Swanson R.A. (2016). Microglial activation induced by the alarmin S100B is regulated by poly(ADP‐ribose) polymerase‐1.. Glia.

[125] Bianchi R., Kastrisianaki E., Giambanco I., Donato R. (2011). S100B protein stimulates microglia migration via RAGE-dependent up-regulation of chemokine expression and release.. J. Biol. Chem..

[126] Zhou S., Zhu W., Zhang Y., Pan S., Bao J. (2018). S100B promotes microglia M1 polarization and migration to aggravate cerebral ischemia.. Inflamm. Res..

[127] Cordeiro J.L., Neves J.D., Nicola F., Vizuete A.F., Sanches E.F., Gonçalves C.A., Netto C.A. (2022). Arundic acid (ONO-2506) attenuates neuroinflammation and prevents motor impairment in rats with intracerebral hemorrhage.. Cell. Mol. Neurobiol..

[128] Freitas-Andrade M., Wang N., Bechberger J.F., De Bock M., Lampe P.D., Leybaert L., Naus C.C. (2019). Targeting MAPK phosphorylation of Connexin43 provides neuroprotection in stroke.. J. Exp. Med..

[129] Retamal M.A., Froger N., Palacios-Prado N., Ezan P., Sáez P.J., Sáez J.C., Giaume C. (2007). Cx43 hemichannels and gap junction channels in astrocytes are regulated oppositely by proinflammatory cytokines released from activated microglia.. J. Neurosci..

[130] Verderio C., Matteoli M. (2001). ATP mediates calcium signaling between astrocytes and microglial cells: modulation by IFN-gamma.. J. Immunol..

[131] Kim Y., Davidson J.O., Green C.R., Nicholson L.F.B., O’Carroll S.J., Zhang J. (2018). Connexins and pannexins in cerebral ischemia.. Biochim. Biophys. Acta Biomembr..

[132] Arbeloa J., Pérez-Samartín A., Gottlieb M., Matute C. (2012). P2X7 receptor blockade prevents ATP excitotoxicity in neurons and reduces brain damage after ischemia.. Neurobiol. Dis..

[133] Sperlágh B., Illes P. (2014). P2X7 receptor: An emerging target in central nervous system diseases.. Trends Pharmacol. Sci..

[134] Chen Y., Luan P., Liu J., Wei Y., Wang C., Wu R., Wu Z., Jing M. (2024). Spatiotemporally selective astrocytic ATP dynamics encode injury information sensed by microglia following brain injury in mice.. Nat. Neurosci..

[135] Goemaere J., Knoops B. (2012). Peroxiredoxin distribution in the mouse brain with emphasis on neuronal populations affected in neurodegenerative disorders.. J. Comp. Neurol..

[136] Asuni A.A., Guridi M., Sanchez S., Sadowski M.J. (2015). Antioxidant peroxiredoxin 6 protein rescues toxicity due to oxidative stress and cellular hypoxia in vitro, and attenuates prion-related pathology in vivo.. Neurochem. Int..

[137] Yu S., Wang X., Lei S., Chen X., Liu Y., Zhou Y., Zhou Y., Wu J., Zhao Y. (2015). Sulfiredoxin-1 protects primary cultured astrocytes from ischemia-induced damage.. Neurochem. Int..

[138] Shanshan Y., Beibei J., Li T., Minna G., Shipeng L., Li P., Yong Z. (2017). Phospholipase A2 of peroxiredoxin 6 plays a critical role in cerebral ischemia/reperfusion inflammatory injury.. Front. Cell. Neurosci..

[139] Peng L., Ji Y., Li Y., You Y., Zhou Y. (2024). PRDX6-iPLA2 aggravates neuroinflammation after ischemic stroke via regulating astrocytes-induced M1 microglia.. Cell Commun. Signal..

[140] Saraiva M., O’Garra A. (2010). The regulation of IL-10 production by immune cells.. Nat. Rev. Immunol..

[141] Piepke M., Clausen B.H., Ludewig P., Vienhues J.H., Bedke T., Javidi E., Rissiek B., Jank L., Brockmann L., Sandrock I., Degenhardt K., Jander A., Roth V., Schädlich I.S., Prinz I., Flavell R.A., Kobayashi Y., Renné T., Gerloff C., Huber S., Magnus T., Gelderblom M. (2021). Interleukin-10 improves stroke outcome by controlling the detrimental Interleukin-17A response.. J. Neuroinflammation.

[142] Bugbee E., Wang A.A., Gommerman J.L. (2023). Under the influence: environmental factors as modulators of neuroinflammation through the IL-10/IL-10R axis.. Front. Immunol..

[143] Li Q., Lan X., Han X., Durham F., Wan J., Weiland A., Koehler R.C., Wang J. (2021). Microglia-derived interleukin-10 accelerates post-intracerebral hemorrhage hematoma clearance by regulating CD36.. Brain Behav. Immun..

[144] Nayak A.R., Kashyap R.S., Purohit H.J., Kabra D., Taori G.M., Daginawala H.F. (2009). Evaluation of the inflammatory response in sera from acute ischemic stroke patients by measurement of IL-2 and IL-10.. Inflamm. Res..

[145] Worthmann H., Tryc A.B., Dirks M., Schuppner R., Brand K., Klawonn F., Lichtinghagen R., Weissenborn K. (2015). Lipopolysaccharide binding protein, interleukin-10, interleukin-6 and C-reactive protein blood levels in acute ischemic stroke patients with post-stroke infection.. J. Neuroinflammation.

[146] Norden D.M., Fenn A.M., Dugan A., Godbout J.P. (2014). TGFβ produced by IL‐10 redirected astrocytes attenuates microglial activation.. Glia.

[147] Wu W., Luo Z., Shen D., Lan T., Xiao Z., Liu M., Hu L., Sun T., Wang Y., Zhang J.N., Zhang C., Wang P., Lu Y., Yang F., Li Q. (2024). IL-10 protects against OPC ferroptosis by regulating lipid reactive oxygen species levels post stroke.. Redox Biol..

[148] Fernandez A.M., Torres-Alemán I. (2012). The many faces of insulin-like peptide signalling in the brain.. Nat. Rev. Neurosci..

[149] Zhu W., Fan Y., Hao Q., Shen F., Hashimoto T., Yang G.Y., Gasmi M., Bartus R.T., Young W.L., Chen Y. (2009). Postischemic IGF-1 gene transfer promotes neurovascular regeneration after experimental stroke.. J. Cereb. Blood Flow Metab..

[150] Suh H.S., Zhao M.L., Derico L., Choi N., Lee S.C. (2013). Insulin-like growth factor 1 and 2 (IGF1, IGF2) expression in human microglia: differential regulation by inflammatory mediators.. J. Neuroinflammation.

[151] Zheng J., Wu H., Wang X., Zhang G., Lu J., Xu W., Xu S., Fang Y., Zhang A., Shao A., Chen S., Zhao Z., Zhang J., Yu J. (2023). Temporal dynamics of microglia-astrocyte interaction in neuroprotective glial scar formation after intracerebral hemorrhage.. J. Pharm. Anal..

[152] Peng J., Yu Z., Xiao R., Hu X., Xia Y. (2023). Exosomal ZEB1 derived from neural stem cells reduces inflammation injury in OGD/R-treated microglia via the GPR30-TLR4-NF-κB Axis.. Neurochem. Res..

[153] de Barrios O., Sanchez-Moral L., Cortés M., Ninfali C., Profitós-Pelejà N., Martínez-Campanario M.C., Siles L., del Campo R., Fernández-Aceñero M.J., Darling D.S., Castells A., Maurel J., Salas A., Dean D.C., Postigo A. (2019). ZEB1 promotes inflammation and progression towards inflammation-driven carcinoma through repression of the DNA repair glycosylase MPG in epithelial cells.. Gut.

[154] Poonaki E., Kahlert U.D., Meuth S.G., Gorji A. (2022). The role of the ZEB1–neuroinflammation axis in CNS disorders.. J. Neuroinflammation.

[155] Bui T., Sequeira J., Wen T.C., Sola A., Higashi Y., Kondoh H., Genetta T. (2009). ZEB1 links p63 and p73 in a novel neuronal survival pathway rapidly induced in response to cortical ischemia.. PLoS One.

[156] Li D., Lang W., Zhou C., Wu C., Zhang F., Liu Q., Yang S., Hao J. (2018). Upregulation of microglial ZEB1 ameliorates brain damage after acute ischemic stroke.. Cell Rep..

[157] Pan Y., Liu Y., Wei W., Yang X., Wang Z., Xin W. (2023). Extracellular vesicles as delivery shippers for noncoding RNA‐based modulation of angiogenesis: Insights from ischemic stroke and cancer.. Small.

[158] Wilson C.M., Belkozhayev A.M., Al-Yozbaki M., George A., Ye Niyazova R., Sharipov K.O., Byrne L.J. (2022). Extracellular vesicles, stem cells and the role of miRNAs in neurodegeneration.. Curr. Neuropharmacol..

[159] Li Z., Song Y., He T., Wen R., Li Y., Chen T., Huang S., Wang Y., Tang Y., Shen F., Tian H.L., Yang G.Y., Zhang Z. (2021). M2 microglial small extracellular vesicles reduce glial scar formation via the miR-124/STAT3 pathway after ischemic stroke in mice.. Theranostics.

[160] Xin W., Pan Y., Wei W., Tatenhorst L., Graf I., Popa-Wagner A., Gerner S.T., Huber S., Kilic E., Hermann D.M., Bähr M., Huttner H.B., Doeppner T.R. (2023). Preconditioned extracellular vesicles from hypoxic microglia reduce poststroke AQP4 depolarization, disturbed cerebrospinal fluid flow, astrogliosis, and neuroinflammation.. Theranostics.

[161] Han P., Mi W.L., Wang Y.Q. (2011). Research progress on interleukin-33 and its roles in the central nervous system.. Neurosci. Bull..

[162] Schmitz J., Owyang A., Oldham E., Song Y., Murphy E., McClanahan T.K., Zurawski G., Moshrefi M., Qin J., Li X., Gorman D.M., Bazan J.F., Kastelein R.A. (2005). IL-33, an interleukin-1-like cytokine that signals via the IL-1 receptor-related protein ST2 and induces T helper type 2-associated cytokines.. Immunity.

[163] Gadani S.P., Walsh J.T., Smirnov I., Zheng J., Kipnis J. (2015). The glia-derived alarmin IL-33 orchestrates the immune response and promotes recovery following CNS injury.. Neuron.

[164] Wicher G., Wallenquist U., Lei Y., Enoksson M., Li X., Fuchs B., Abu Hamdeh S., Marklund N., Hillered L., Nilsson G., Forsberg-Nilsson K. (2017). Interleukin-33 promotes recruitment of microglia/macrophages in response to traumatic brain injury.. J. Neurotrauma.

[165] Yang D., Sun Y., Lin D., Li S., Zhang Y., Wu A., Wei C. (2024). Interleukin-33 ameliorates perioperative neurocognitive disorders by modulating microglial state.. Neuropharmacology.

[166] Nguyen P.T., Dorman L.C., Pan S., Vainchtein I.D., Han R.T., Nakao-Inoue H., Taloma S.E., Barron J.J., Molofsky A.B., Kheirbek M.A., Molofsky A.V. (2020). Microglial remodeling of the extracellular matrix promotes synapse plasticity.. Cell.

[167] Gao Y., Ma L., Luo C., Wang T., Zhang M., Shen X., Meng H., Ji M., Wang Z., Chen X., Tao L. (2017). IL-33 exerts neuroprotective effect in mice intracerebral hemorrhage model through suppressing inflammation/apoptotic/autophagic pathway.. Mol. Neurobiol..

[168] Xie D., Liu H., Xu F., Su W., Ye Q., Yu F., Austin T.J., Chen J., Hu X. (2021). IL33 (Interleukin 33)/ST2 (Interleukin 1 Receptor-Like 1) axis drives protective microglial responses and promotes white matter integrity after stroke.. Stroke.

[169] Yang Y., Liu H., Zhang H., Ye Q., Wang J., Yang B., Mao L., Zhu W., Leak R.K., Xiao B., Lu B., Chen J., Hu X. (2017). ST2/IL-33-dependent microglial response limits acute ischemic brain injury.. J. Neurosci..

[170] Han S., Lone M.A., Schneiter R., Chang A. (2010). Orm1 and Orm2 are conserved endoplasmic reticulum membrane proteins regulating lipid homeostasis and protein quality control.. Proc. Natl. Acad. Sci. USA.

[171] Luo Z., Lei H., Sun Y., Liu X., Su D.F. (2015). Orosomucoid, an acute response protein with multiple modulating activities.. J. Physiol. Biochem..

[172] Wan J.J., Wang P.Y., Zhang Y., Qin Z., Sun Y., Hu B.H., Su D.F., Xu D.P., Liu X. (2019). Role of acute‐phase protein ORM in a mice model of ischemic stroke.. J. Cell. Physiol..

[173] Jo M., Kim J.H., Song G.J., Seo M., Hwang E.M., Suk K. (2017). Astrocytic orosomucoid-2 modulates microglial activation and neuroinflammation.. J. Neurosci..

[174] Liu X., Lv X., Liu Z., Zhang M., Leng Y. (2022). MircoRNA-29a in astrocyte-derived extracellular vesicles suppresses brain ischemia reperfusion injury via TP53INP1 and the NF-κB/NLRP3 axis.. Cell. Mol. Neurobiol..

[175] Long X., Yao X., Jiang Q., Yang Y., He X., Tian W., Zhao K., Zhang H. (2020). Astrocyte-derived exosomes enriched with miR-873a-5p inhibit neuroinflammation via microglia phenotype modulation after traumatic brain injury.. J. Neuroinflammation.

[176] Qian Y., Li X., Li G., Liu H., Li Q., Liu X., Zhang Y., He Z., Zhao Y., Fan H. (2024). Astrocyte-derived exosomal miR-148a-3p suppresses neuroinflammation and restores neurological function in traumatic brain injury by regulating the microglial phenotype.. eNeuro.

[177] Hayakawa K., Esposito E., Wang X., Terasaki Y., Liu Y., Xing C., Ji X., Lo E.H. (2016). Transfer of mitochondria from astrocytes to neurons after stroke.. Nature.

[178] Jung J.E., Sun G., Bautista Garrido J., Obertas L., Mobley A.S., Ting S.M., Zhao X., Aronowski J. (2020). The mitochondria-derived peptide humanin improves recovery from intracerebral hemorrhage: Implication of mitochondria transfer and microglia phenotype change.. J. Neurosci..

[179] Tashiro R., Bautista-Garrido J., Ozaki D., Sun G., Obertas L., Mobley A.S., Kim G.S., Aronowski J., Jung J.E. (2022). Transplantation of astrocytic mitochondria modulates neuronal antioxidant defense and neuroplasticity and promotes functional recovery after intracerebral hemorrhage.. J. Neurosci..

[180] Perez-de-Puig I., Miró-Mur F., Ferrer-Ferrer M., Gelpi E., Pedragosa J., Justicia C., Urra X., Chamorro A., Planas A.M. (2015). Neutrophil recruitment to the brain in mouse and human ischemic stroke.. Acta Neuropathol..

[181] Soto-Díaz K., Juda M.B., Blackmore S., Walsh C., Steelman A.J. (2020). TAK1 inhibition in mouse astrocyte cultures ameliorates cytokine‐induced chemokine production and neutrophil migration.. J. Neurochem..

[182] Qian H., Zhang H.N., Gao T., Wang X.S., Wang X., Yu M.Y., Li M.K., Huang J. (2024). Upregulation of TRPC1 in microglia promotes neutrophil infiltration after ischemic stroke.. Brain Res. Bull..

[183] Cuartero M.I., Ballesteros I., Moraga A., Nombela F., Vivancos J., Hamilton J.A., Corbí Á.L., Lizasoain I., Moro M.A. (2013). N2 neutrophils, novel players in brain inflammation after stroke: Modulation by the PPARγ agonist rosiglitazone.. Stroke.

[184] Kim Y.R., Kim Y.M., Lee J., Park J., Lee J.E., Hyun Y.M. (2020). Neutrophils return to bloodstream through the brain blood vessel after crosstalk with microglia during LPS-induced neuroinflammation.. Front. Cell Dev. Biol..

[185] Cai W., Liu S., Hu M., Huang F., Zhu Q., Qiu W., Hu X., Colello J., Zheng S.G., Lu Z. (2020). Functional dynamics of neutrophils after ischemic stroke.. Transl. Stroke Res..

[186] Otxoa-de-Amezaga A., Miró-Mur F., Pedragosa J., Gallizioli M., Justicia C., Gaja-Capdevila N., Ruíz-Jaen F., Salas-Perdomo A., Bosch A., Calvo M., Márquez-Kisinousky L., Denes A., Gunzer M., Planas A.M. (2019). Microglial cell loss after ischemic stroke favors brain neutrophil accumulation.. Acta Neuropathol..

[187] Zhao X., Ting S.M., Liu C.H., Sun G., Kruzel M., Roy-O’Reilly M., Aronowski J. (2017). Neutrophil polarization by IL-27 as a therapeutic target for intracerebral hemorrhage.. Nat. Commun..

[188] Miró-Mur F., Pérez-de-Puig I., Ferrer-Ferrer M., Urra X., Justicia C., Chamorro A., Planas A.M. (2016). Immature monocytes recruited to the ischemic mouse brain differentiate into macrophages with features of alternative activation.. Brain Behav. Immun..

[189] Fang W., Zhai X., Han D., Xiong X., Wang T., Zeng X., He S., Liu R., Miyata M., Xu B., Zhao H. (2018). CCR2-dependent monocytes/macrophages exacerbate acute brain injury but promote functional recovery after ischemic stroke in mice.. Theranostics.

[190] Park J., Kim J.Y., Kim Y.R., Huang M., Chang J.Y., Sim A.Y., Jung H., Lee W.T., Hyun Y.M., Lee J.E. (2021). Reparative system arising from CCR2(+) monocyte conversion attenuates neuroinflammation following ischemic stroke.. Transl. Stroke Res..

[191] Gliem M., Krammes K., Liaw L., van Rooijen N., Hartung H.P., Jander S. (2015). Macrophage-derived osteopontin induces reactive astrocyte polarization and promotes re-establishment of the blood brain barrier after ischemic stroke.. Glia.

[192] Ortega S.B., Noorbhai I., Poinsatte K., Kong X., Anderson A., Monson N.L., Stowe A.M. (2015). Stroke induces a rapid adaptive autoimmune response to novel neuronal antigens.. Discov. Med..

[193] Dolati S., Ahmadi M., Khalili M., Taheraghdam A.A., Siahmansouri H., Babaloo Z., Aghebati-Maleki L., Jadidi-Niaragh F., Younesi V., Yousefi M. (2018). Peripheral Th17/Treg imbalance in elderly patients with ischemic stroke.. Neurol. Sci..

[194] Shi Z., Yu P., Lin W.J., Chen S., Hu X., Chen S., Cheng J., Liu Q., Yang Y., Li S., Zhang Z., Xie J., Jiang J., He B., Li Y., Li H., Xu Y., Zeng J., Huang J., Mei J., Cai J., Chen J., Wu L.J., Ko H., Tang Y. (2023). Microglia drive transient insult-induced brain injury by chemotactic recruitment of CD8+ T lymphocytes.. Neuron.

[195] Ito M., Komai K., Mise-Omata S., Iizuka-Koga M., Noguchi Y., Kondo T., Sakai R., Matsuo K., Nakayama T., Yoshie O., Nakatsukasa H., Chikuma S., Shichita T., Yoshimura A. (2019). Brain regulatory T cells suppress astrogliosis and potentiate neurological recovery.. Nature.

[196] Shu L., Xu C., Yan Z.Y., Yan Y., Jiang S.Z., Wang Y.R. (2019). Post-stroke microglia induce sirtuin2 expression to suppress the anti-inflammatory function of infiltrating regulatory T cells.. Inflammation.

[197] Arunachalam P., Ludewig P., Melich P., Arumugam T.V., Gerloff C., Prinz I., Magnus T., Gelderblom M. (2017). CCR6 (CC chemokine receptor 6) is essential for the migration of detrimental natural interleukin-17–producing γδ T cells in stroke.. Stroke.

[198] Mo Y., Xu W., Fu K., Chen H., Wen J., Huang Q., Guo F., Mo L., Yan J. (2022). The dual function of microglial polarization and its treatment targets in ischemic stroke.. Front. Neurol..

[199] Yang S., Wang H., Yang Y., Wang R., Wang Y., Wu C., Du G. (2019). Baicalein administered in the subacute phase ameliorates ischemia-reperfusion-induced brain injury by reducing neuroinflammation and neuronal damage.. Biomed. Pharmacother..

[200] Wang Q., Lv C., Sun Y., Han X., Wang S., Mao Z., Xin Y., Zhang B. (2018). The role of alpha-lipoic acid in the pathomechanism of acute ischemic stroke.. Cell. Physiol. Biochem..

[201] Shi H., Zheng K., Su Z., Su H., Zhong M., He X., Zhou C., Chen H., Xiong Q., Zhang Y. (2016). Sinomenine enhances microglia M2 polarization and attenuates inflammatory injury in intracerebral hemorrhage.. J. Neuroimmunol..

[202] Chen W., Guo C., Huang S., Jia Z., Wang J., Zhong J., Ge H., Yuan J., Chen T., Liu X., Hu R., Yin Y., Feng H. (2020). MitoQ attenuates brain damage by polarizing microglia towards the M2 phenotype through inhibition of the NLRP3 inflammasome after ICH.. Pharmacol. Res..

[203] Wang W., Redecker C., Yu Z.Y., Xie M.J., Tian D.S., Zhang L., Bu B.T., Witte O.W. (2008). Rat focal cerebral ischemia induced astrocyte proliferation and delayed neuronal death are attenuated by cyclin-dependent kinase inhibition.. J. Clin. Neurosci..

[204] Sun C., Lin L., Yin L., Hao X., Tian J., Zhang X., Ren Y., Li C., Yang Y. (2022). Acutely inhibiting AQP4 With TGN-020 improves functional outcome by attenuating edema and peri-infarct astrogliosis after cerebral ischemia.. Front. Immunol..

[205] Yang Y., Yi J., Pan M., Hu B., Duan H. (2021). Edaravone alleviated propofol‐induced neural injury in developing rats by BDNF/TrkB pathway.. J. Cell. Mol. Med..

[206] Ni X.C., Wang H.F., Cai Y.Y., Yang D., Alolga R.N., Liu B., Li J., Huang F.Q. (2022). Ginsenoside Rb1 inhibits astrocyte activation and promotes transfer of astrocytic mitochondria to neurons against ischemic stroke.. Redox Biol..

[207] Song X., Gong Z., Liu K., Kou J., Liu B., Liu K. (2020). Baicalin combats glutamate excitotoxicity via protecting glutamine synthetase from ROS-induced 20S proteasomal degradation.. Redox Biol..

[208] Cao J., Dong L., Luo J., Zeng F., Hong Z., Liu Y., Zhao Y., Xia Z., Zuo D., Xu L., Tao T. (2021). Supplemental N‐3 polyunsaturated fatty acids limit A1‐specific astrocyte polarization via attenuating mitochondrial dysfunction in ischemic stroke in mice.. Oxid. Med. Cell. Longev..

[209] Jin W.N., Shi S.X.Y., Li Z., Li M., Wood K., Gonzales R.J., Liu Q. (2017). Depletion of microglia exacerbates postischemic inflammation and brain injury.. J. Cereb. Blood Flow Metab..

[210] Marino Lee S., Hudobenko J., McCullough L.D., Chauhan A. (2021). Microglia depletion increase brain injury after acute ischemic stroke in aged mice.. Exp. Neurol..

[211] Li T., Zhao J., Gao H. (2022). Depletion of Arg1-positive microglia/macrophages exacerbates cerebral ischemic damage by facilitating the inflammatory response.. Int. J. Mol. Sci..

[212] Zeyen T., Noristani R., Habib S., Heinisch O., Slowik A., Huber M., Schulz J.B., Reich A., Habib P. (2020). Microglial-specific depletion of TAK1 is neuroprotective in the acute phase after ischemic stroke.. J. Mol. Med..

[213] Li M., Li Z., Ren H., Jin W.N., Wood K., Liu Q., Sheth K.N., Shi F.D. (2017). Colony stimulating factor 1 receptor inhibition eliminates microglia and attenuates brain injury after intracerebral hemorrhage.. J. Cereb. Blood Flow Metab..

[214] Wang Q., Ding H., Chen S., Liu X., Deng Y., Jiang W., Li Y., Huang L., Han Y., Wen M., Wang M., Zeng H. (2020). Hypertonic saline mediates the NLRP3/IL‐1β signaling axis in microglia to alleviate ischemic blood‐brain barrier permeability by downregulating astrocyte‐derived VEGF in rats.. CNS Neurosci. Ther..

[215] Zheng J., Lu J., Mei S., Wu H., Sun Z., Fang Y., Xu S., Wang X., Shi L., Xu W., Chen S., Yu J., Liang F., Zhang J. (2021). Ceria nanoparticles ameliorate white matter injury after intracerebral hemorrhage: Microglia-astrocyte involvement in remyelination.. J. Neuroinflammation.

[216] Takei R., Nakashima M., Gotoh M., Endo M., Hashimoto K., Miyamoto Y., Murakami-Murofushi K. (2023). 2-carba-cyclic phosphatidic acid modulates astrocyte-to-microglia communication and influences microglial polarization towards an anti-inflammatory phenotype.. Neurosci. Lett..

[217] Kano S., Choi E.Y., Dohi E., Agarwal S., Chang D.J., Wilson A.M., Lo B.D., Rose I.V.L., Gonzalez S., Imai T., Sawa A. (2019). Glutathione S-transferases promote proinflammatory astrocyte-microglia communication during brain inflammation.. Sci. Signal..

[218] Saviano A., Henderson N.C., Baumert T.F. (2020). Single-cell genomics and spatial transcriptomics: Discovery of novel cell states and cellular interactions in liver physiology and disease biology.. J. Hepatol..

[219] Smajić S., Prada-Medina C.A., Landoulsi Z., Ghelfi J., Delcambre S., Dietrich C., Jarazo J., Henck J., Balachandran S., Pachchek S., Morris C.M., Antony P., Timmermann B., Sauer S., Pereira S.L., Schwamborn J.C., May P., Grünewald A., Spielmann M. (2022). Single-cell sequencing of human midbrain reveals glial activation and a Parkinson-specific neuronal state.. Brain.

[220] Akbar M., MacDonald L., Crowe L.A.N., Carlberg K., Kurowska-Stolarska M., Ståhl P.L., Snelling S.J.B., McInnes I.B., Millar N.L. (2021). Single cell and spatial transcriptomics in human tendon disease indicate dysregulated immune homeostasis.. Ann. Rheum. Dis..

